# Synthesis and Characterization of Novel 2-(1,2,3-Triazol-4-yl)-4,5-dihydro-1*H*-pyrazol-1-yl)thiazoles and 2-(4,5-Dihydro-1*H*-pyrazol-1-yl)-4-(1*H*-1,2,3-triazol-4-yl)thiazoles

**DOI:** 10.3390/molecules27248904

**Published:** 2022-12-14

**Authors:** Benson M. Kariuki, Bakr F. Abdel-Wahab, Hanan A. Mohamed, Mohamed S. Bekheit, Gamal A. El-Hiti

**Affiliations:** 1 School of Chemistry, Cardiff University, Main Building, Park Place, Cardiff CF10 3AT, UK; 2 Applied Organic Chemistry Department, Chemical Industries Research Institute, National Research Centre, Dokki, Giza 12622, Egypt; 3 Department of Pesticide Chemistry, National Research Centre, Dokki, Giza 12622, Egypt; 4 Department of Optometry, College of Applied Medical Sciences, King Saud University, Riyadh 11433, Saudi Arabia

**Keywords:** 2-(1,2,3-triazol-4-yl)-4,5-dihydro-1*H*-pyrazol-1-yl)thiazoles, 1,2,3-triazoles, pyrazoline, 1,3-thiazoles, synthesis, single crystal X-ray

## Abstract

Reactions of 1-(5-methyl)-1*H*-1,2,3-triazol-4-yl)ethan-1-ones and benzaldehydes in ethanol under basic conditions gave the corresponding chalcones. Reactions of the chalcones combined with thiosemicarbazide in dry ethanol containing sodium hydroxide afforded the corresponding pyrazolin-*N*-thioamides. Reactions of the synthesized pyrazolin-*N*-thioamides and several ketones (namely, ethyl 2-chloro-3-oxobutanoate, 2-bromoacetylbenzofuran, and hydrazonoyl chloride) gave the corresponding novel 2-(1,2,3-triazol-4-yl)-4,5-dihydro-1*H*-pyrazol-1-yl)thiazoles in high yields (77–90%). Additionally, 2-(4,5-dihydro-1*H*-pyrazol-1-yl)-4-(1*H*-1,2,3-triazol-4-yl)thiazoles were obtained in high yields (84–87%) from reactions with *N*-pyrazoline-thioamides and 4-bromoacetyl-1,2,3-triazoles under basic conditions. The structures of six of the newly synthesized heterocycles were confirmed by X-ray crystallography.

## 1. Introduction

Heterocycles containing nitrogen and sulfur have received great attention due to their pharmacological and industrial applications [1,2]. Pyrazoles present antimicrobial, anti-inflammatory, antihypertensive, antipyretic, antioxidant, analgesic, antidepressant, anticancer, and antidiabetic activities [3,4,5]. Naturally occurring thiazole compounds also have antibacterial, anti-inflammatory, antifungal, antihypertensive, neuroleptic, and antimalarial properties [6,7,8,9]. In addition, 1,2,3-triazoles display anti-HIV, antimicrobial, antiviral, and antiproliferative effects [10,11,12,13]. Derivatives of 1,2,3-triazole have been used as insecticides, fungicides, and plant growth regulators [14,15]. The synthesis of heterocycles containing pyrazole, thiazole, and triazole moieties is therefore of interest to both the academic and industrial communities.

Recent synthetic methods used to produce pyrazoles involve, for example, the cycloaddition of *N*-isocyanoiminotriphenylphosphorane in the presence of silver carbonate [16], oxidative condensations of carbonyl compounds (aldehydes and ketones) and hydrazine monohydrochloride [17], oxidative cyclization of β,γ-unsaturated hydrazones [18], and the reaction of enaminones and hydrazines in the presence of iodine as a catalyst [19]. For the production of thiazoles, the most recent synthetic procedures involve reactions of aldehydes and amines in the presence of sulfur and copper chloride [20], enaminoesters and fluorodibromoamides in the presence of sulfur [21], primary amines and α-nitro epoxides in the presence of potassium thiocyanate [22], and α-oxodithioesters and tosylmethyl isocyanide in the presence of potassium hydroxide [23]. The synthesis of 1,2,4-triazoles includes reactions of *N*-tosylhydrazones with sodium azide [24], cyclization of 4-toluenesulfonyl hydrazines and methyl ketones in the presence of iodine and 1-aminopyridinium iodide [25], α-ketoacetals and amines [26], and cycloaddition of azides, propiolic acids, and arylboronic acids in the presence of different catalysts [27].

Pyrazolyltriazoles can be synthesized using different procedures. For example, reactions of 2-bromoketones and pyrazole-1-carbothioamides or thiazolyl hydrazines and dicarbonyl or β-ketonitriles led to the production of pyrazolyltriazoles [28]. Triazolylthiazoles can be produced from the cyclization of Schiff bases containing a triazole moiety in the presence of mercaptoacetic acids, or from carbohydrazides containing thiazole units and isothiocyanates or carbon disulfides, followed by cyclization and elimination of hydrogen sulfide [28]. Recently, we have reported the synthesis and crystal structures of novel heterocycles containing thiazole, pyrazoline, and 1,2,4-triazole moieties [29,30,31] as part of our long-term interest in new biologically active heterocycles [32,33,34]. In the current work, we report the synthesis of 2-(1,2,3-triazol-4-yl)-4,5-dihydro-1*H*-pyrazol-1-yl)thiazoles and 2-(4,5-dihydro-1*H*-pyrazol-1-yl)-4-(1*H*-1,2,3-triazol-4-yl)thiazoles using simple procedures and their structure characterization. The synthesized compounds are complex in structure since they contain various heterocyclic rings and substituents. No related heterocycles are reported in the literature and therefore direct comparison is not possible.

## 2. Results and Discussion

### Synthesis of Novel Heterocycles

**Scheme 1:**
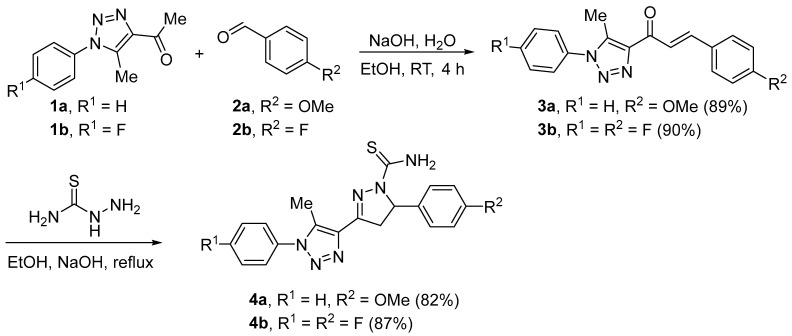
Synthesis of compounds **3a,b** and **4a,b**.

Reactions of 1-(5-methyl)-1*H*-1,2,3-triazol-4-yl)ethan-1-ones **1a,b** (R^1^ = H, F) and benzaldehydes **2a,b** (R^2^ = OMe, F) in ethanol (EtOH) under basic conditions gave chalcones **3a,b**. The reactions of **3a** and **3b** and thiosemicarbazide in dry ethanol containing sodium hydroxide (NaOH) afforded the corresponding pyrazolin-*N*-thioamides **4a** and **4b**, respectively (Scheme 1).

**Figure 1:**
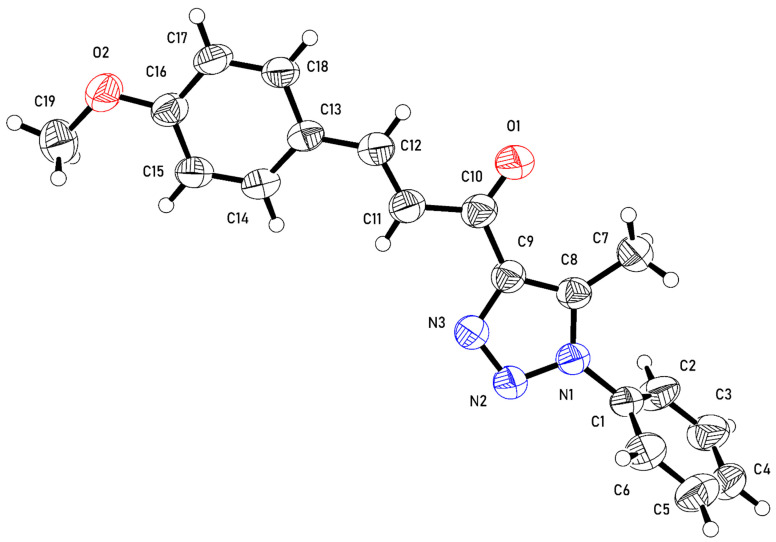
An ortep representation of **3a** showing 50% probability atomic displacement ellipsoids.

**Figure 2:**
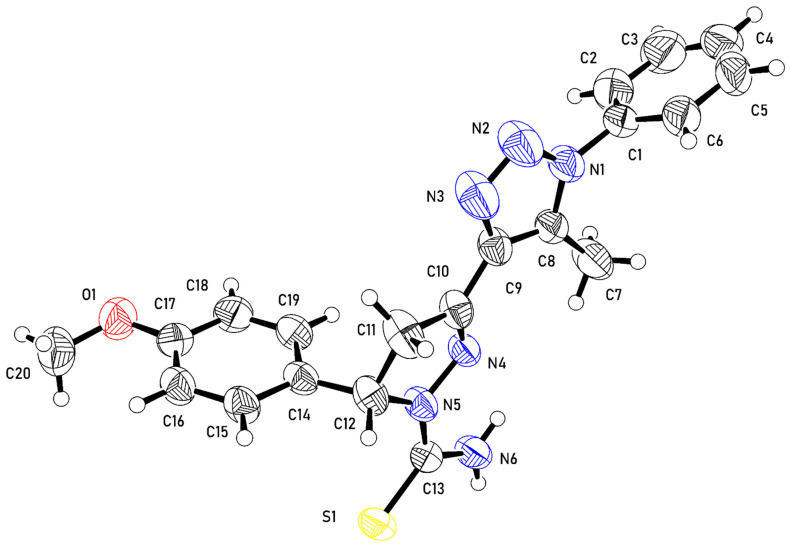
An ortep representation of **4a** showing 50% probability atomic displacement ellipsoids.

The chemical structures of **3a** and **4a** were confirmed by single crystal X-ray diffraction, as shown in Figure 1 and Figure 2, respectively. The CH=CH protons in **3a** appeared as two doublets (*J* = 16.0 Hz) at 7.79 and 7.86 ppm in its ^1^H NMR spectrum, while the CH=CH carbons appeared at 120.9 and 143.7 ppm in the ^13^C NMR spectrum. For **4a**, the ^1^H NMR spectrum showed two doublets at 13.6 (*J* = 18.2 Hz) and 5.86 (*J* = 8.1 Hz) that correspond to the CH_2_ protons of pyrazoline. The C=S carbon appeared highly down-field (176.5 ppm) in the ^13^C NMR spectrum. For details, see the Supplementary Materials.

For X-ray crystal structure determination, single crystals were obtained following the crystallization of the synthesized heterocycles using dimethylformamide (DMF). The molecule of **3a** (Figure 1) is constructed from phenyl **A** (C1–C6), methyltriazole **B** (C7–C9, N1–N3), propanal **C** (C10–C12, O1) and methoxybenzene **D** (C13–C19, O2) groups. Except for the phenyl group (**A**), the molecule is nearly planar as shown by the twist angles **B**/**C** and **C**/**D** of 4.23(19)° and 9.82(18)°, respectively. Regarding the phenyl group, twist angle **A**/**B** is 66.96(7)°.

The molecule of **4a** (Figure 2) contains phenyl **A** (C1–C6), methyltriazole **B** (C7–C9, N1–N3), pyrazole **C** (C10–C12, N4, N5), methoxybenzene **D** (C14–C20, O1) and methanethioamide **E** (C13, S1, N6) groups. In the crystal, the pyrazole ring (**C**) is distorted from planarity with C12 diverging from the least squares plane of the rest of the atoms by 0.235(5)Å. The methanethioamide (**E**) and pyrazole (**C**) groups are roughly coplanar with a **C**/**E** twist angle of 10.67(21)°. The methyltriazole (**B**) and pyrazole (**C**) groups are also almost coplanar with a **B**/**C** twist angle of 15.35(24)°. The phenyl (**A**) and methoxybenzene (**D**) groups are significantly twisted from this plane with twist angles **A**/**B** and **C**/**D** of 78.71(12)° and 87.02(11)°, respectively. Hydrogen bonding of type N–H...N (N6…N4 = 3.117(3)Å, N6-H6B…N4 = 162.1°) and N–H..S (N6…S1 = 3.506(3)Å, N6–H6A…S1= 138.7°) is observed in the crystal structure. For more details, see the Supplementary Materials.

**Figure 3:**
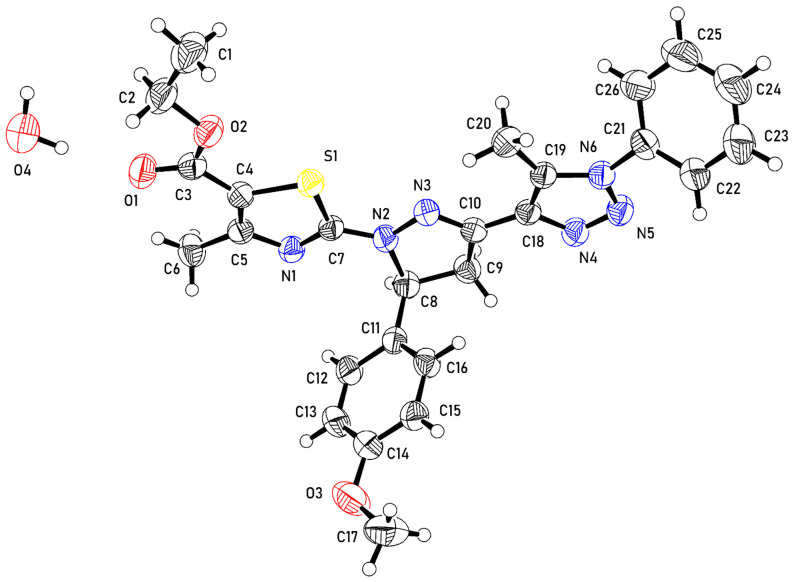
An ortep representation of **8** showing 50% probability atomic displacement ellipsoids.

**Figure 4:**
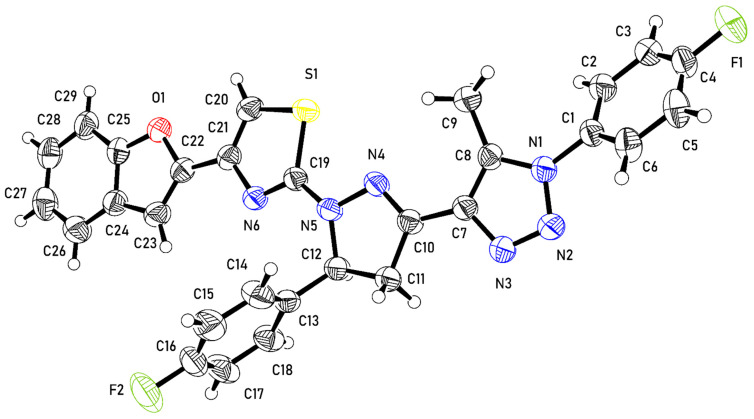
An ortep representation of **9** showing 50% probability atomic displacement ellipsoids.

**Figure 5:**
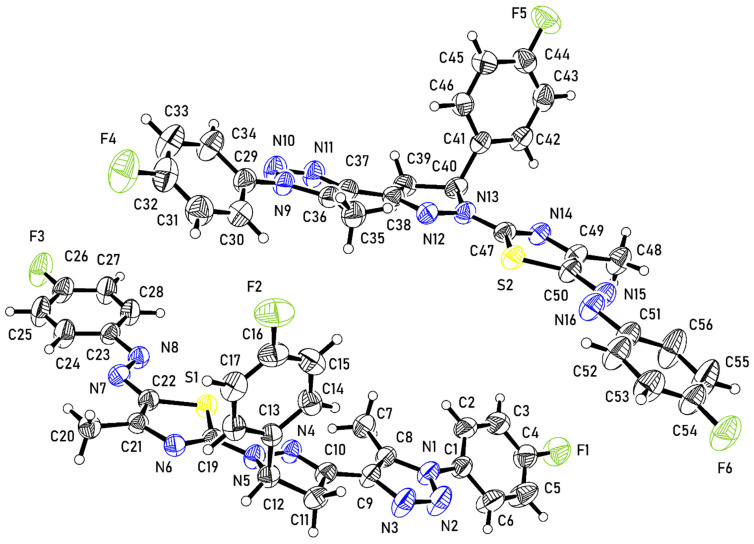
An ortep representation of **10** showing 50% probability atomic displacement ellipsoids.

**Scheme 2:**
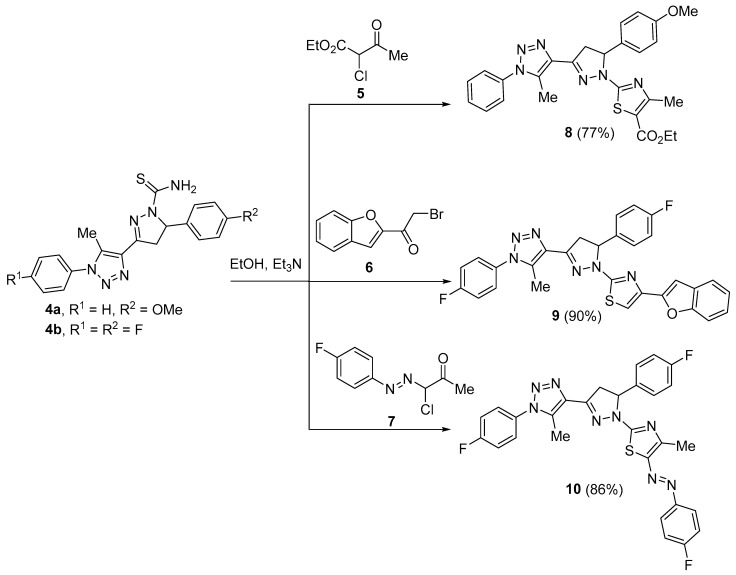
Synthesis of heterocycles **8**–**10**.

The reaction of thioamide **4a** and ethyl 2-chloro-3-oxobutanoate (**5**) in EtOH and in the presence of triethylamine (Et_3_N) gave ethyl 2-(5-(4-methoxyphenyl)-3-(5-methyl-1-phenyl-1*H*-1,2,3-triazol-4-yl)-4,5-dihydro-1*H*-pyrazol-1-yl)-4-methylthiazole-5-carboxylate (**8**) in 77% yield (Scheme 2). Similarly, the reaction of **4b** and 2-acetylbenzofuran (**6**) or 1-chloro-1-((4-fluorophenyl)diazenyl)propan-2-one (**7**) gave 4-(benzofuran-2-yl)-2-(5-(4-fluorophenyl)-3-(1-(4-fluorophenyl)-5-methyl-1*H*-1,2,3-triazol-4-yl)-4,5-dihydro-1*H*-pyrazol-1-yl)thiazole (**9**) or 2-(5-(4-fluorophenyl)-3-(1-(4-fluorophenyl)-5-methyl-1*H*-1,2,3-triazol-4-yl)-4,5-dihydro-1*H*-pyrazol-1-yl)-5-((4-fluorophenyl)diazenyl)-4-methylthiazole (**10**) in 90 or 86% yield, respectively (Scheme 2). The structures of **8**–**10** were confirmed by single crystal X-ray diffraction (Figure 3, Figure 4 and Figure 5).

The ^1^H NMR spectrum of compound **8** showed two characteristic double doublets (*J* = 4.8 Hz) that appeared at 3.37 and 4.42 ppm, corresponding to the CH_2_ protons of the pyrazolinyl moiety. In addition, it showed a double doublet (*J* = 4.8 and 16.4 Hz) that appeared at 5.70 ppm corresponding to the pyrazolinyl proton at position 5. The ^13^C NMR spectrum of **8** showed two high-field signals at 162.3 and 165.3 ppm that correspond to the carbonyl carbon and C2 of the thiazolyl moiety, respectively. For compound **9**, the pyrazoline protons appeared as three double doublets of one proton each at 3.43 (*J* = 6.2 and 18.1 Hz), 4.18 (*J* = 11.9 and 18.1 Hz), and 5.68 (*J* = 6.2 and 11.9 Hz). The ^13^C NMR spectra of **9** and **10** showed the coupling between the fluorine atoms and the carbons (C2–C6) of the aryl rings.

The crystal structure of **8** also contains water molecules (Figure 3). The molecule of **8** contains ethylformate **A** (C1–C3, O1, O2), methylthiazole **B** (C4–C7, N1, S1), pyrazole **C** (C8–C10, N2, N3), methyltriazole **D** (C18–C20, N4–N6), phenyl **E** (C21–C26), and methoxybenzene **F** (C11–C17, O3) groups. The ethylformate (**A**) is flat with a maximum deviation from the least squares plane of 0.050(3) Å. The pyrazole (**C**) ring is slightly deformed from planarity with C8 being 0.356(4)Å from the plane of the other atoms of the ring. Groups **A**–**D** are coplanar as indicated by twist angles **A**/**B**, **B**/**C**, **C**/**D** of 4.76(14)°, 1.71(13)°, 0.72(13)°, respectively. Phenyl ring (**E**) and methoxybenzene (**F**) groups diverge from the **A**-**D** plane with twist angles **D**/**E** and **C**/**F** of 44.03(7)° and 85.82(7)°, respectively. The water molecule forms two hydrogen bonds with the carbonyl oxygen (O4…O1 = 2.904(3), O4–H41…O1 = 171(4)°) and triazole nitrogen (O4…N4 = 3.128(3)Å, O4–H42…N4 = 162(4)°) of compound **8**.

The molecule of **9** is made of two fluorobenzene **A** (F1, C1–C6), **F** (F2, C13–C18), methyltriazole **B** (C7–C9, N1–N3), pyrazole **C** (C10–C12, N4, N5), thiazole **D** (C19–C21, N6, S1) and benzofuran **E** (C22–C29, O1) groups. The pyrazole ring (**C**) is in envelope conformation with atom C12 located 0.309(4)Å from the plane through the rest of the atoms. The **B**–**E** backbone of the molecule is essentially planar with twist angles **B**/**C**, **C**/**D**, **D**/**E** of 2.99(20)°, 10.18(17)° and 2.45(12)°, respectively. The fluorobenzene rings (**A** and **F**) are twisted from this plane with **A**/**B** and **C**/**F** angles of 42.65(8)° and 81.12(7)°, respectively.

The crystal structure of **10** contains two independent molecules (Figure 5). The first molecule consists of three fluorobenzene groups, namely **A1** (C1–C6, F1), **E1** (C23–C28, F3) and **F1** (C13–C18, F2), as well as methyltriazole **B1**, (C7–C9, N1–N3), pyrazole **C1** (C10–C12, N4, N5), methylthiazole **D1** (C19–C22, N6, S1) and diazene (N7, N8) groups. The central part of the molecule (**B1**–**E1**) is almost planar as indicated by the twist angles **B1**/**C1**, **C1**/**D1** and **D1**/**E1** of 3.80(13)°, 3.71(12)° and 9.98(11)°, respectively. Two of the fluorophenyl groups (**A1** and **F1**) are twisted from this plane with **A1**/**B1** and **C1**/**F1** angles of 31.76(10)° and 89.53(10)°, respectively.

The second independent molecule has three fluorobenzene **A2**, (C29–C34, F3), **E2**, (C51–C56, F6) and **F2**, (C41–C46, F5) moieties along with methyltriazole **B2**, (C35–C37, N9–N11), pyrazole **C2** (C38–C40, N12, N13), methylthiazole **D2** (C47–C50, N14, S2) and diazene (N15, N16) groups. Similar geometry to that for the first molecule is observed for the second molecule. The middle part of the molecule (**B2**–**E2**) is almost planar with twist angles **B2**/**C2**, **C2**/**D2** and **D2**/**E2** of 3.38(12)°, 6.48(12)° and 8.18(11)°, respectively. Two of the fluorophenyl groups (**A2** and **F2**) are oriented substantially away from this plane with twist angles **A2**/**B2** and **C2**/**F2** of 31.85(8)° and 82.19(8)°, respectively. In both independent molecules, the pyrazole rings (**C1** and **C2**) are in envelop conformation with C12 deviating from the least squares planes of the rest of the atoms by 0.324(3)Å for the first molecule and C40 deviating by 0.271(3)Å for the second molecule.

**Scheme 3:**
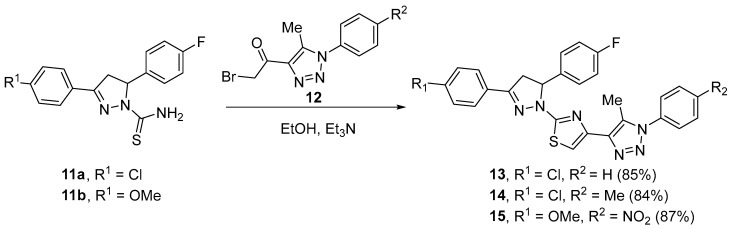
Synthesis of compounds **13**–**15**.

**Figure 6:**
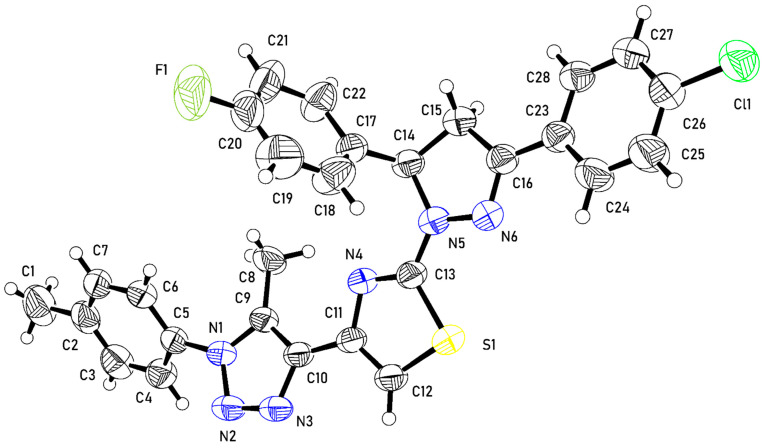
An ortep representation of **14** showing 50% probability atomic displacement ellipsoids.

2-(4,5-Dihydro-1*H*-pyrazol-1-yl)-4-(1*H*-1,2,3-triazol-4-yl)thiazoles **13a**–**13c** were synthesized in excellent yields from reactions of *N*-pyrazoline-thioamides **11a,b** and 4-bromoacetyl-1,2,3-triazole derivatives **12a**–**12c** in anhydrous EtOH and in the presence of Et_3_N under reflux conditions to give the corresponding heterocycles **13**–**15** (Scheme 3). The chemical structure of **14** was confirmed by single crystal X-ray diffraction (Figure 6). The ^1^H NMR spectra of **13**–**15** showed three double doublets for the pyrazoline protons that appeared at the 3.24–3.28, 3.98–3.99, and 5.54–3.54 ppm regions. The ^13^C NMR spectra of **13–15** showed the C2 of the thiazole ring at very low-field in the region of 165.4–165.8 ppm.

The molecule of **14** (Figure 6) comprises methylbenzene **A** (C1–C7), methyltriazole **B** (C8–C10, N1–N3), thiazole **C** (C11–C13, N4, S1), pyrazole **D** (C14–C16, N5, N6), chlorobenzene **E** (C23–C28, Cl1), fluorobenzene **F** (C17–C22, F1). The pyrazole ring is slightly distorted from planarity, with C14 deviating from the plane of the rest of the atoms by 0.186(4)Å. The backbone of the molecule (**B**–**E**) is almost planar with twist angles **B**/**C**, **C**/**D** and **D**/**E** of 15.85(12)°, 8.19(18)° and 7.91(17)°, respectively. Rings **A** and **F** deviate from the planar segment of the molecule with twist angles **A**/**B** and **D**/**F** of 48.46(9)° and 74.59(8)°, respectively.

## 3. Materials and Methods

### 3.1. General

Sodium hydroxide (98%), 4-fluorobenzaldehyde (99%), 4-methoxybenzaldehyde (98%), thiosemicarbazide (98%), triethylamine (99%), and solvents were obtained from different sources, including Merck (Gillingham, UK) and Thermo Fisher Scientific (Waltham, MA, USA). Melting points were determined using an Electrothermal melting point apparatus (Cole-Parmer, Illinois, IL, USA. The NMR spectra (δ in ppm and *J* in Hz) were recorded in dimethyl sulfoxide (DMSO-d_6_) using a JEOL NMR 500 MHz spectrometer (Tokyo, Japan) at 500 MHz for the ^1^H and 25 MHz for the ^13^C NMR measurements. Microanalyses of carbon, hydrogen, and nitrogen were carried out using a CHNS-932 (LECO) Vario elemental analyzer. Compounds **1** [35], **3b** [36], **4b** [29], **6** [36], **7** [37], **11** [38,39], and **12** [40] were prepared based on procedures in the literature.

### 3.2. Synthesis of Chalcones ***3a,b***

A mixture of **1a** or **1b** (12 mmol) and **2a** or **2b** (12 mmol) in EtOH (50 mL) was added slowly to a solution of NaOH (0.5 g, 12.2 mmol) in water (10 mL). The mixture was stirred at 25 °C for 4 h and the solid obtained was filtrated, washed with cold water, dried, and recrystallized from EtOH to give pure **3a** or **3b**.

#### 3.2.1. (*E*)-3-(4-Methoxyphenyl)-1-(5-methyl-1-phenyl-1H-1,2,3-Triazol-4-yl)prop-2-en-1-one (**3a**)

Yield: 89%, mp 144–145 °C. ^1^H NMR (ppm): 2.59 (s, 3H, Me), 3.79 (s, 3H, OMe), 7.00 (d, 8.6 Hz, 2H, Ar), 7.61–7.63 (m, 5H, Ph), 7.71 (d, 8.6 Hz, 2H, Ar), 7.79 (d, 16.0 Hz, 1H, CH), 7.86 (d, 16.0 Hz, 1H, CH). ^13^C NMR (ppm): 10.4 (Me), 55.9 (OMe), 115.1 (C3/C5 of Ar), 120.9 (CH), 126.0 (C2/C6 of Ph), 127.6 (C1 of Ar), 130.2 (C2/C6 of Ar), 130.6 (C4 of Ph), 131.1 (C3/C5 of Ph), 135.6 (C5 of triazolyl), 139.01 (C1 of Ph), 143.5 (C4 of triazolyl), 143.7 (CH), 162.0 (C4 of Ar), 183.8 (C=O). Anal. Calcd. for C_19_H_17_N_3_O_2_ (319.13): C, 71.46; H, 5.37; N, 13.16. Found: C, 71.53, H, 5.66, N, 13.23%.

#### 3.2.2. (*E*)-3-(4-Fluorophenyl)-1-(1-(4-fluorophenyl)-5-methyl-1H-1,2,3-triazol-4-yl)prop-2-en-1-one (**3b**)

Yield: 90%, mp 170–172 °C (lit. 168–170 [36]). The spectroscopic data of **3b** agreed with those reported.

### 3.3. Synthesis of Pyrazolin-N-Thioamides ***4a,b***

To a suspension of chalcone **3a** or **3b** (10 mmol) and NaOH (1.0 g, 25 mmol) in EtOH (50 mL), thiosemicarbazide (1.1 g, 12 mmol) was added. The mixture was refluxed for 6 h and the solid obtained on cooling was filtered, washed with EtOH, and dried to give **4a** or **4b**.

#### 3.3.1. 5-(4-Methoxyphenyl)-3-(5-methyl-1-phenyl-1H-1,2,3-triazol-4-yl)-4,5-dihydro-1H-pyrazole-1-carbothioamide (**4a**)

Yield: 82%, mp 183–185 °C. ^1^H NMR (ppm) 2.96 (s, 3H, Me), 3.16 (d, 18.2 Hz, 1H, 1H of CH_2_ of pyrazoline), 3.95 (s, 3H, OMe), 3.98 (dd, 8.1 and 18.2 Hz, 1H, pyrazolinyl), 5.86 (d, 8.1 Hz, 1H, 1H of CH_2_ of pyrazoline), 6.85 (d, 8.6 Hz, 2H, Ar), 7.06 (d, 8.6 Hz, 2H, Ar), 7.57–7.59 (m, 5H, Ph), 8.03 (s, 1H, exch., NH). ^13^C NMR (ppm): 10.7 (Me), 40.5 (C4 of pyrazolinyl), 55.6 (OMe), 61.8 (C5 of pyrazolinyl), 114.4 (C3/C5 of Ar), 125.9 (C2/C6 of Ph), 127.2 (C2/C6 of Ar), 130.3 (C3/C5 of Ph), 130.5 (C4 of Ph), 135.1 (C1 of Ar), 135.3 (C1 of Ph), 135.9 (C5 of triazolyl), 137.5 (C4 of triazolyl), 150.8 (C3 of pyrazolinyl), 158.8 (C4 of Ar), 176.5 (C=S). Anal. Calcd. for C_20_H_20_N_6_OS (392.14): C, 61.21; H, 5.14; N, 21.41. Found: C, 61.47, H, 5.42, N, 21.66%.

#### 3.3.2. 5-(4-Fluorophenyl)-3-(1-(4-fluorophenyl)-5-methyl-1H-1,2,3-triazol-4-yl)-4,5-dihydro-1H-pyrazole-1-carbothioamide (**4b**)

Yield: 87%, mp 230–232 °C (lit. 229–231 [29]). The spectroscopic data of **4b** agreed with those reported.

### 3.4. Synthesis of Compounds ***8**–**10***

To a suspension of **4a** or **4b** (2 mmol) in EtOH (15 mL) and Et_3_N (0.2 mL, **5** (0.16 g, 1 mmol), **6** (0.24 g, 1 mmol) or **7** (0.22 g, 1 mmol) was added. The mixture was heated under reflux for 2 h and the solid obtained on cooling was collected by suction filtration, washed with EtOH, and recrystallized from DMF to give the corresponding heterocycle **8**, **9**, or **10**, respectively.

#### 3.4.1. Ethyl 2-(5-(4-Methoxyphenyl)-3-(5-methyl-1-phenyl-1H-1,2,3-triazol-4-yl)-4,5-dihydro-1H-pyrazol-1-yl)-4-methylthiazole-5-carboxylate (**8**)

Yield: 77%, mp 145–146 °C. ^1^H NMR (ppm, Hz): 1.33 (t, 7.0 Hz, 3H, *Me*CH_2_), 2.38 (s, 3H, Me), 2.63 (s, 3H, Me), 3.37 (d, 4.8 Hz, 1H, 1H of CH_2_ of pyrazoline), 3.42 (d, 4.8 Hz, 1H, 1H of CH_2_ of pyrazolinyl), 3.73 (s, 3H, OMe), 4.21 (q, 7.0 Hz, 2H, Me*CH_2_*), 5.70 (dd, 4.8 and 16.4 Hz, 1H, pyrazolinyl), 6.92 (d, 8.8 Hz, 2H, Ar), 7.22 (d, 8.8 Hz, 2H, Ar), 7.65–7.68 (m, 5H, Ar). ^13^C NMR (ppm): 10.6 (Me), 14.8 (Me), 18.0 (Me), 45.2 (C4 of pyrazolinyl), 55.5 (OMe), 60.4 (C5 of pyrazolinyl), 62.1 (Me*C*H_2_), 110.8 (C3/C5 of Ar), 114.6 (C5 of thiazolyl), 125.7 (C2/C6 of Ph), 127.7 (C2/C6 of Ar), 130.2 (C3/C5 of Ph), 130.5 (C4 of Ph), 133.4 (C3 of pyrazolinyl), 134.8 (C1 of Ar), 135.8 (C1 of Ph), 137.2 (C5 of triazolyl), 150.5 (C4 of triazolyl), 159.2 (C4 of thiazolyl), 159.7 (C4 of Ar), 162.3 (C=O), 165.3 (C2 of thiazolyl). Anal. Calcd. for C_26_H_26_N_6_O_3_S (502.17): C, 62.13; H, 5.21; N, 16.72. Found: C, 62.29, H, 5.33, N, 16.85%.

#### 3.4.2. 4-(Benzofuran-2-yl)-2-(5-(4-fluorophenyl)-3-(1-(4-fluorophenyl)-5-methyl-1H-1,2,3-triazol-4-yl)-4,5-dihydro-1H-pyrazol-1-yl)thiazole (**9**)

Yield: 90%, mp 215–216 °C. ^1^H NMR (ppm): 2.59 (s, 3H, Me), 3.43 (dd, 6.2 and 18.1 Hz, 1H, 1H of CH_2_ of pyrazoline), 4.18 (dd, 11.9 and 18.1 Hz, 1H, 1H of CH_2_ of pyrazoline), 5.68 (dd, 6.2 and 11.9 Hz, 1H, pyrazolinyl), 6.88 (s, 1H, thiazolyl), 7.18–7.22 (m, 3H, Ar), 7.26 (t, 7.6 Hz, 1H, benzofuranyl), 7.30 (s, 1H, benzofuranyl), 7.46–7.53 (m, 5H, Ar), 7.61 (d, 7.6 Hz, 1H, Ar), 7.71–7.73 (m, 2H, Ar). ^13^C NMR (ppm): 10.5 (Me), 45.0 (C4 of pyrazolinyl), 63.0 (C5 of pyrazolinyl), 103.1 (C3 of benzofuranyl), 107.4 (C5 of thiazolyl), 114.5 (C7 of benzofuranyl), 116.0 (d, 21.5 Hz, C3/C5 of Ar), 117.3 (d, 23.9 Hz, C3/C5 of Ar), 121.9 (C4 of benzofuranyl), 123.8 (C5 of benzofuranyl), 125.2 (C6 of benzofuranyl), 128.3 (d, 9.6 Hz, C2/C6 of Ar), 128.9 (C4a of benzofuranyl), 129.4 (d, 8.4 Hz, C2/C6 of Ar), 132.3 (C5 of triazolyl), 134.7 (C4 of triazolyl), 137.5 (C1 of Ar), 138.0 (C1 of Ar), 142.6 (C4 of thiazolyl), 148.7 (C2 of benzofuranyl), 152.4 (C7a of benzofuranyl), 154.6 (C3 of pyrazolinyl), 161.6 (d, 115.7 Hz, C-4 of Ar), 163.4 (d, 130.0 Hz, C4 of Ar), 165.7 (C2 of thiazolyl). Anal. Calcd. for C_29_H_20_F_2_N_6_OS (538.13): C, 64.67; H, 3.74; N, 15.60. Found: C, 64.77, H, 3.89, N, 15.88%.

#### 3.4.3. 2-(5-(4-Fluorophenyl)-3-(1-(4-fluorophenyl)-5-methyl-1H-1,2,3-triazol-4-yl)-4,5-dihydro-1H-pyrazol-1-yl)-5-((4-fluorophenyl)diazenyl)-4-methylthiazole (**10**)

Yield: 86%, mp 222–223 °C. ^1^H NMR (ppm): 2.44 (s, 3H, Me), 2.58 (s, 3H, Me), 3.40 (dd, 4.3 and 18.1 Hz, 1H, 1H of CH_2_ of pyrazoline), 4.20 (dd, 11.4 and 18.6 Hz, 1H, 1H of CH_2_ of pyrazoline), 5.83 (dd, 4.3 and 11.4 Hz 1H, pyrazolinyl), 7.18 (app. t, 8.6 Hz, 2H, Ar), 7.24 (app. t, 8.6 Hz, 2H, Ar), 7.33 (m, 2H, Ar), 7.48 (app. t, 8.6 Hz, 2H, Ar), 7.67–7.71 (m, 4H, Ar). ^13^C NMR (ppm): 10.6 (Me), 16.5 (Me), 45.2 (C4 of pyrazolinyl), 61.8 (C5 of pyrazolinyl), 116.3 (d, 23.8 Hz, C3/C5 of Ar), 117.2 (d, 23.0 Hz, C3/C5 of Ar), 117.4 (d, 23.2 Hz, C3/C5 of Ar), 124.0 (d, 8.4 Hz, C2/C6 of Ar), 128.3 (d, 8.4 Hz, C2/C6 of Ar), 128.4 (d, 8.4 Hz, C2/C6 of Ar), 132.2 (C3 of pyrazolinyl), 135.5 (C5 of thiazolyl), 137.1 (C5 of triazolyl), 137.5 (C4 of thiazolyl), 140.9 (C1 of Ar), 149.5 (C1 of Ar), 151.6 (C1 of Ar), 158.7 (C4 of triazolyl), 161.3 (d, 83.5 Hz, C4 of Ar), 162.6 (d, 124.0 Hz, C4 of Ar), 163.8 (C2 of thiazolyl), 164.5 (d, 112.1 Hz, C4 of Ar). Anal. calcd. for C_28_H_21_F_3_N_8_S (558.15): C, 60.21; H, 3.79; N, 20.06. Found: C, 60.38, H, 3.91, N, 20.21%.

### 3.5. Synthesis of Compounds ***13**–**15***

To a suspension of compound **11a** or **11b** (2 mmol) in EtOH (15 mL) and Et_3_N (0.2 mL), appropriate bromoketones **12a**–**12c** (2 mmol) were added. The mixture was heated under reflux for 2 h and the solid obtained on cooling was collected by filtration, washed with EtOH, and recrystallized from DMF to give the corresponding compound **13**–**15**.

#### 3.5.1. 2-(3-(4-Chlorophenyl)-5-(4-fluorophenyl)-4,5-dihydro-1H-pyrazol-1-yl)-4-(5-methyl-1-phenyl-1H-1,2,3-triazol-4-yl)thiazole (**13**)

Yield: 85%, mp 237–238 °C. ^1^H NMR (ppm): 2.18 (s, 3H, Me), 3.28 (dd, 7.7 and 17.8 Hz, 1H, 1H of CH_2_ of pyrazoline), 3.99 (dd, 11.8 and 17.8 Hz, 1H, 1H of CH_2_ of pyrazoline), 5.59 (dd, 7.7 and 11.8 Hz, 1H, pyrazolinyl), 7.13 (app. t, 9.0 Hz, 2H, Ar), 7.25 (s, 1H, 1H, thiazolyl), 7.38 (app. t, 9.0 Hz, 2H, Ar), 7.50–7.58 (m, 7H, Ph and Ar), 7.76 (d, 2H, 8.1 Hz, H2/H6 of Ph). ^13^C NMR (ppm): 9.7 (Me), 43.8 (C4 of pyrazolinyl), 64.8 (C5 of pyrazolinyl), 105.8 (C5 of thiazolyl), 115.9 (d, 21.5 Hz, C3/C5 of Ar), 125.8 (C2/C6 of Ph), 128.7 (C2/C6 of Ar), 129.1 (d, 8.4 Hz, C2/C6 of Ar), 129.4 (C3/C5 of Ph), 130.0 (C3/C5 of Ar), 130.1 (C4 of Ph), 130.3 (C5 of triazolyl), 131.1 (C3 of pyrazolinyl), 135.1 (C1 of Ar), 136.4 (C4 of Ar), 138.5 (C1 of Ph), 140.2 (C4 of triazolyl), 143.9 1 (C4 of thiazolyl), 152.5 (C1 of Ar), 161.9 (d, 240.8 Hz, C4 of Ar), 165.5 (C2 of thiazolyl). Anal. Calcd. for C_27_H_20_ClFN_6_S (514.11): C, 62.97; H, 3.91; N, 16.32. Found: C, 63.11, H, 4.08, N, 16.49%.

#### 3.5.2. 2-(3-(4-Chlorophenyl)-5-(4-fluorophenyl)-4,5-dihydro-1H-pyrazol-1-yl)-4-(5-methyl-1-(4-tolyl)-1H-1,2,3-triazol-4-yl)thiazole (**14**)

Yield: 84%, mp 252–253 °C. ^1^H NMR (ppm): 2.15 (s, 3H, Me), 2.37 (s, 3H, Me), 3.28 (dd, 7.7 and 17.7 Hz, 1H, 1H of CH_2_ of pyrazoline), 3.98 (dd, 11.9 and 17.7 Hz, 1H, 1H of CH_2_ of pyrazoline), 5.58 (dd, 7.7 and 11.7 Hz, 1H, pyrazolinyl), 7.12 (app. t, 8.6 Hz, 2H, Ar), 7.24 (s, 1H, thiazolyl), 7.35–7.41 (m, 6H, Ar and Ph), 7.50 (d, 8.6 Hz, 2H, Ar), 7.75 (d, 8.6 Hz, 2H, Ar). ^13^C NMR (ppm): 9.7 (Me), 21.3 (Me), 43.8 (C4 of pyrazolinyl), 64.8 (C5 of pyrazolinyl), 105.7 (C5 of thiazolyl), 115.9 (d, 21.5 Hz, C3/C5 of Ar), 125.6 (C2/C6 of Ar), 128.6 (C2/C6 of Ar), 129.1 (d, 8.4 Hz, C2/C6 of Ar), 129.4 (C3/C5 of Ar), 130.4 (C5 of triazolyl), 130.5 (C3/C5 of Ar), 131.1 (C4 of triazolyl), 133.9 (C3 of pyrazolinyl), 134.0 (C1 of Ar), 135.1 (C4 of Ar), 139.8 (C1 of Ar), 140.1 (C4 of thiazolyl), 144.0 (C4 of Ar), 152.5 (C1 of Ar), 162.0 (d, 243.2 Hz, C4 of Ar), 165.4 (C2 of thiazolyl). Anal. Calcd. for C_28_H_22_ClFN_6_S (528.13): C, 63.57; H, 4.19; N, 15.89. Found: C, 63.70, H, 4.14, N, 15.99.

#### 3.5.3. 2-(5-(4-Fluorophenyl)-3-(4-methoxyphenyl)-4,5-dihydro-1H-pyrazol-1-yl)-4-(5-methyl-1-(4-nitrophenyl)-1H-1,2,3-triazol-4-yl)thiazole (**15**)

Yield: 87%, mp 287–288 °C. ^1^H NMR (ppm): 2.28 (s, 3H, Me), 3.24 (dd, 7.6 and 17.6 Hz, 1H, 1H of CH_2_ of pyrazoline), 3.77 (s, 3H, OMe), 3.98 (dd, 11.4 and 17.7 Hz, 1H, 1H of CH_2_ of pyrazoline), 5.54 (dd, 7.6 and 11.4 Hz, 1H, pyrazolinyl), 7.00 (d, 8.7 Hz, 2H, Ar), 7.13 (app. t, 8.6 Hz, 2H, Ar), 7.25 (s, 1H, thiazolyl), 7.37 (m, 2H, Ar), 7.69 (d, 8.6 Hz, 2H, Ar), 7.89 (d, 9.0 Hz, 2H, Ar), 7.39 (d, 8.6 Hz, 2H, Ar). ^13^C NMR (ppm): 9.9 (Me), 44.2 (C4 of pyrazolinyl), 55.9 (OMe), 64.5 (C5 of pyrazolinyl), 105.9 (C5 of thiazolyl), 114.8 (C3/C5 of Ar), 115.9 (d, 21.5 Hz, C3/C5 of Ar), 123.9 (C3/C5 of Ar), 125.5 (C2/C6 of Ar), 126.4 (C2/C6 of Ar), 127.6 (C3 of pyrazolinyl), 128.7 (C2/C6 of Ar), 129.0 (d, 8.3 Hz, C2/C6 of Ar), 131.4 (C5 of triazolyl), 138.7 (C4 of thiazolyl), 140.8 (C1 of Ar), 141.2 (C4 of triazolyl), 143.4 (C1 of Ar), 147.9 (C4 of Ar), 153.5 (C1 of Ar), 160.1.2 (d, 46.5 Hz, C4 of Ar), 165.8 (C2 of thiazolyl). Anal. Calcd. for C_28_H_22_FN_7_O_3_S (555.15): C, 60.53; H, 3.99; N, 17.65. Found: C, 60.69, H, 3.89, N, 17.81%.

### 3.6. Crystal Structure Determination

Single-crystal X-ray diffraction data were collected at room temperature on an Agilent SuperNova Dual Atlas diffractometer with mirror-monochromated Cu or Mo radiation. SHELXS [41] and SHELXL [42] were used for crystal structure solution and refinement. Anisotropic displacement parameters were used in the refinement for non-hydrogen atoms. A riding model with idealized geometry was used for the hydrogen atoms and Uiso(H) were set at 1.2 of 1.5 the values for the atom to which the hydrogen atoms are bonded. Crystal data, data collection and structure refinement details are summarized in Table 1. The crystal structures have been deposited in the Cambridge Structural Database under reference CCDC Numbers 2220613–2220618.

## 4. Conclusions

Novel 2-(1,2,3-triazol-4-yl)-4,5-dihydro-1*H*-pyrazol-1-yl)thiazoles and 2-(4,5-dihydro-1*H*-pyrazol-1-yl)-4-(1*H*-1,2,3-triazol-4-yl)thiazoles were synthesized in high yields using facile methods and their chemical structures were established using spectral data and the single crystal X-ray diffraction.

## Figures and Tables

**Table 1:** Data collection and refinement data for compounds **3a**, **4a**, **8**–**10**, and **14**.

	3a	4a	8	9	10	14
MF	C_19_H_17_N_3_O_2_	C_20_H_20_N_6_OS	C_26_H_28_N_6_O_4_S	C_29_H_20_F_2_N_6_OS	C_28_H_21_F_3_N_8_S	C_28_H_22_ClFN_6_S
FW	319.35	392.48	520.60	538.57	558.59	529.02
T (K)	296 (2)	296 (2)	296 (2)	298 (2)	296 (2)	293 (2)
λ (Å)	0.71073	1.54184	0.71073	0.71073	0.71073	0.71073
System	Triclinic	Orthorhombic	Monoclinic	Triclinic	Triclinic	Monoclinic
Space group	$P\bar{1}$	Pca2_1_	P2_1_/n	$P\bar{1}$	$P\bar{1}$	P2_1_/c
A (Å)	8.7987 (7)	23.0527 (11)	17.7187 (7)	6.5793 (4)	11.4945 (5)	12.7331 (8)
b (Å)	9.4342 (11)	11.8887 (5)	7.6878 (4)	13.1589 (8)	14.8707 (6)	21.2977 (9)
c (Å)	10.6006 (13)	7.2229 (3)	19.1203 (9)	16.0368 (12)	16.2570 (6)	9.7424 (5)
α (°)	85.681 (10)	90	90	65.959 (6)	96.807 (3)	90
β (°)	72.823 (9)	90	94.160 (4)	82.697 (5)	95.999 (4)	105.329 (6)
γ (°)	79.304 (9)	90	90	87.701 (5)	106.731 (4)	90
Volume (Å^3^)	825.89 (16)	1979.56 (15)	2597.7 (2)	1257.58 (15)	2614.20 (19)	2548.0 (2)
Z	2	4	4	2	4	4
D (Mg/m^−3^)	1.284	1.317	1.331	1.422	1.419	1.379
μ/(mm^−1^)	0.085	1.641	0.169	0.180	0.180	0.269
F (000)	336	824	1096	556	1152	1096
Crystal size (mm^3^)	0.480 × 0.170 × 0.120	0.326 × 0.092 × 0.045	0.329 × 0.144 × 0.062	0.275 × 0.142 × 0.091	0.434 × 0.156 × 0.065	0.278 × 0.122 × 0.119
Reflections collected	6864	9173	24844	11051	25295	24815
Independent reflections	3935	3397	6550	5939	11910	6378
R (int)	0.0369	0.0222	0.0616	0.0341	0.0327	0.0282
Goodness-of-fit on F^2^	1.057	1.050	1.030	1.031	1.012	1.036
R1 [I > 2sigma(I)]	0.0627	0.0369	0.0573	0.0538	0.0508	0.0487
wR2 [I > 2sigma(I)]	0.1639	0.0955	0.1380	0.1156	0.1233	0.1194
R1 (all data)	0.0928	0.0400	0.1006	0.0868	0.0945,	0.0834
wR2 (all data)	0.1935	0.0989	0.1640	0.1386	0.1523	0.1395
Largest diff. peak and hole (e.Å^−3^)	0.231/−0.196	0.137/−0.170	0.275/−0.245	0.197/−0.283	0.221/−0.265	0.234/−0.316

MF: molecular formula, FW: formula weight, T: temperature, λ: wavelength, D: calculated density, and μ: absorption coefficient.

## Data Availability

Data are contained within the article and the Supplementary Materials.

## Supplementary Materials

The following are available online at https://www.mdpi.com/article/10.3390/molecules27248904/s1, ^1^H and ^13^C NMR and IR spectra for the synthesized compounds and CIF and checkcif reports for compounds **3a**, **4a**, **8**–**10**, and **14**.

## Sample Availability

Samples of the synthesized compounds are available from the authors.

## References

[1] Sharma P.K., Amin A., Kumar M. (2020). A review: Medicinally important nitrogen sulphur containing heterocycles. Open Med. Chem. J..

[2] De S., Aamna B., Sahu R., Parida S., Behera S.K., Dan A.K. (2022). Seeking heterocyclic scaffolds as antivirals against dengue virus. Eur. J. Med. Chem..

[3] Karrouchi K., Radi S., Ramli Y., Taoufik J., Mabkhot Y.N., Al-aizari F.A., Ansar M. (2018). Synthesis and pharmacological activities of pyrazole derivatives: A review. Molecules.

[4] Ebenezer O., Shapi M., Tuszynski J.A. (2022). A review of the recent development in the synthesis and biological evaluations of pyrazole derivatives. Biomedicines.

[5] Naim M.J., Alam O., Nawaz F., Alam M.J., Alam P. (2016). Current status of pyrazole and its biological activities. J. Pharm. Bioallied. Sci..

[6] Chhabria M.T., Patel S., Modi P., Brahmkshatriya P.S. (2016). Thiazole: A review on chemistry, synthesis and therapeutic importance of its derivatives. Curr. Top. Med. Chem..

[7] Nayak S., Gaonkar S.L. (2019). A review on recent synthetic strategies and pharmacological importance of 1,3-thiazole derivatives. Mini Rev. Med. Chem..

[8] Leoni A., Locatelli A., Morigi R., Rambaldi M. (2014). Novel thiazole derivatives: A patent review (2008–2012; Part 1). Expert. Opin. Ther. Pat..

[9] Alia S.H., Sayed A.R. (2021). Review of the synthesis and biological activity of thiazoles. Synth. Commun..

[10] 1Akter M., Rupa K., Anbarasan P. (2022). 1,2,3-Triazole and its analogues: New surrogates for diazo compounds. Chem. Rev..

[11] Alam M.M. (2022). 1,2,3-Triazole hybrids as anticancer agents: A review. Arch. Pharm..

[12] 1Wen X., Zhou Y., Zeng J., Liu X. (2020). Recent development of 1,2,4-triazole-containing compounds as anticancer agents. Curr. Top. Med. Chem..

[13] Bozorov K., Zhao J., Aisa H.A. (2019). 1,2,3-Triazole-containing hybrids as leads in medicinal chemistry: A recent overview. Bioorg. Med. Chem..

[14] Thabit T.M.A., Abdelkareem E.M., Bouqellah N.A., Shokr S.A. (2021). Triazole fungicide residues and their inhibitory effect on some trichothecenes mycotoxin excretion in wheat grains. Molecules.

[15] Ijaz M., Ul-Allah S., Sher A., Sattar A., Ahmad W., Rehim A., Honermeier B. (2022). Chapter 19—Effects of triazoles, strobilurins, and trinexapac ethyl on growth and development of crop plants under stress conditions. J. Plant Growth Regul..

[16] Yi F., Zhao W., Wang Z., Bi X. (2019). Silver-mediated [3 + 2] cycloaddition of alkynes and *N*-isocyanoiminotriphenylphosphorane: Access to monosubstituted pyrazoles. Org. Lett..

[17] Lellek V., Chen C.-Y., Yang W., Liu J., Ji X., Faessler R. (2018). An efficient synthesis of substituted pyrazoles from one-pot reaction of ketones, aldehydes, and hydrazine monohydrochloride. Synlett.

[18] Fan Z., Feng J., Hou Y., Rao M., Cheng J. (2020). Copper-catalyzed aerobic cyclization of β,γ-unsaturated hydrazones with concomitant C═C bond cleavage. Org. Lett..

[19] Guo H., Tian L., Liu Y., Wan J.-P. (2022). DMSO as a C1 source for [2 + 2 + 1] pyrazole ring construction via metal-free annulation with enaminones and hydrazines. Org. Lett..

[20] Wang X., Qiu X., Wei J., Liu J., Song S., Wang W., Jiao N. (2018). Cu-catalyzed aerobic oxidative sulfuration/annulation approach to thiazoles via multiple Csp^3^–H bond cleavage. Org. Lett..

[21] Ma X., Yu X., Huang H., Zhou Y., Song Q. (2020). Synthesis of thiazoles and isothiazoles via three-component reaction of enaminoesters, sulfur, and bromodifluoroacetamides/esters. Org. Lett..

[22] Zhu Y., Chen W., Zhao D., Zhang G., Yu Y. (2019). One-pot three-component strategy for polysubstituted 2-aminothiazoles via ring opening of α-nitro epoxides. Synthesis.

[23] Kiran K.R., Swaroop T.R., Rajeev N., Anil S.M., Rangappa J.S., Sadashiva M.P. (2020). Cyclization of active methylene isocyanides with α-oxodithioesters induced by base: An expedient synthesis of 4-methylthio/ethoxycarbonyl-5-acylthiazoles. Synthesis.

[24] Shu W.-M., Zhang X.-F., Zhang X.-X., Li M., Wang A.-J., Wu A.-X. (2019). Metal-free cascade [4 + 1] cyclization access to 4-aryl-*NH*-1,2,3-triazoles from *N*-tosylhydrazones and sodium azide. J. Org. Chem..

[25] Huang C., Geng X., Zhao P., Zhou Y., Yu X.-X., Wang L.-S., Wu Y.-D., Wu A.-X. (2021). Direct synthesis of 4-aryl-1,2,3-triazoles via I_2_-promoted cyclization under metal- and azide-free conditions. J. Org. Chem..

[26] Zehnder L.R., Hawkins J.M., Sutton S.C. (2020). One-pot, metal- and azide-free synthesis of 1,2,3-triazoles from α-ketoacetals and amines. Synlett.

[27] Wang X.-X., Xin Y., Li Y., Xia W.-J., Zhou B., Ye R.-R., Li Y.-M. (2020). Copper-catalyzed decarboxylative cycloaddition of propiolic acids, azides, and arylboronic acids: Construction of fully substituted 1,2,3-triazoles. J. Org. Chem..

[28] Abdel-Wahab B.F., Mohamed H.A., Farahat A.A., Dawood K.M. (2011). Synthetic accesses to azolylthiazoles. Heterocycles.

[29] Kariuki B.M., Abdel-Wahab B.F., El-Hiti G.A. (2021). Synthesis and structural characterization of isostructural 4-(4-aryl)-2-(5-(4-fluorophenyl)-3-(1-(4-fluorophenyl)-5-methyl-1*H*-1,2,3-triazol-4-yl)-4,5-dihydro-1*H*-pyrazol-1-yl)thiazoles. Crystals.

[30] El-Hiti G.A., Abdel-Wahab B.F., Baashen M., Hegazy A.S., Kariuki B.M. (2017). Crystal structure of (*E*)-5-((4-chlorophenyl)diazenyl)-2-(5-(4-fluorophenyl)-3-(thiophen-2-yl)-4,5-dihydro-1*H*-pyrazol-1-yl)-4-methylthiazole, C_23_H_17_ClFN_5_S_2_. Z. Für Krist.-New Cryst. Struct..

[31] Ghabbour H.A., Abdel-Wahab B.F., Alamri M., Al-Omar M.A., El-Hiti G.A. (2017). Crystal structure of 2-(5-(4-fluorophenyl)-3-p-tolyl-4,5-dihydro-1*H*-pyrazol-1-yl)-4-(5-methyl-1-*p*-tolyl-1*H*-1,2,3-triazol-4-yl)thiazole, C_29_H_25_FN_6_S. Z. Für Krist.-New Cryst. Struct..

[32] Mohamed H.A., Abdel-Wahab B.F., Sabry E., Kariuki B.M., El-Hiti G.A. (2022). Synthesis and antimicrobial activity of 2,5-*bis*(pyrazol-3-yl or triazol-4-yl)-1,3,4-oxadiazoles. Heterocycles.

[33] Abdel-Wahab B.F., Mohamed H.A., Farahat A.A., Kariuki B.M., El-Hiti G.A. (2022). reactivity of 4-bromoacetyl-1,2,3-triazoles towards amines and phenols: Synthesis and antimicrobial activity of novel heterocycles. Heterocycles.

[34] Abdel-Wahab B.F., Khidre R.E., Mohamed H.A., El-Hiti G.A. (2017). A simple process for the synthesis of novel pyrazolyltriazole and dihydropyrazolylthiazole derivatives as antimicrobial agents. Arab. J. Sci. Eng..

[35] Kamalraj V.R., Senthil S., Kannan P. (2008). One-pot synthesis and the fluorescent behavior of 4-acetyl-5-methyl-1,2,3-triazole regioisomers. J. Mol. Struct..

[36] Shriner R.L., Anderson J. (1939). Derivatives of coumaran. VI. Reduction of 2-acetobenzofuran and its derivatives. J. Am. Chem. Soc..

[37] Biere H., Schroeder E., Ahrens H., Kapp J.F., Boettcher I. (1982). Non-steroidal antiinflammatory agents. 5. Antiinflammatory pyrazole acetic acids. Eur. J. Med. Chem..

[38] Chimenti F., Carradori S., Secci D., Bolasco A., Bizzarri B., Chimenti P., Granese A., Yanez M., Orallo F. (2010). Synthesis and inhibitory activity against human monoamine oxidase of N1-thiocarbamoyl-3,5-di(hetero)aryl-4,5-dihydro-(1*H*)-pyrazole derivatives. Eur. J. Med. Chem..

[39] Abdel-Wahab B.F., Mohamed H.A., Ng S.W., Tiekink E.R.T. (2013). 3-(4-Chlorophenyl)-5-(4-fluorophenyl)-4,5-dihydro-1*H*-pyrazole-1-carbothioamide. Acta Cryst..

[40] Bunev A.S., Trushkova Y.O., Ostapenko G.I., Statsyuk V.E., Peregudov A.S. (2014). Synthesis of 4-(1*H*-1,2,3-Triazol-4-yl)-1,3-thiazole-2-amine derivatives. Chem. Heterocycl. Compd..

[41] Sheldrick G.M. (2008). A short history of SHELX. Acta Crystallogr. A.

[42] Sheldrick G.M. (2015). Crystal structure refinement with SHELXL. Acta Crystallogr. C.

