# Supplementary material for: Synthesis and Characterization of Novel 2-(1,2,3-Triazol-4-yl)-4,5-dihydro-1H-pyrazol-1-yl)thiazoles and 2-(4,5-Dihydro-1H-pyrazol-1-yl)-4-(1H-1,2,3-triazol-4-yl)thiazoles
Source: Molecules. 2022 Dec 14;27(24):8904. doi: 10.3390/molecules27248904 (PMC9786072; doi:10.3390/molecules27248904)

checkCIF/PLATON report

Structure factors have been supplied for datablock(s) bmk1936b

THIS REPORT IS FOR GUIDANCE ONLY. IF USED AS PART OF A REVIEW PROCEDURE FOR PUBLICATION, IT SHOULD NOT REPLACE THE EXPERTISE OF AN EXPERIENCED CRYSTALLOGRAPHIC REFEREE.

No syntax errors found. CIF dictionary Interpreting this report

Datablock: bmk1936b

```
Bond precision:   C-C = 0.0035 A          Wavelength=0.71073

Cell:             a=17.7187(7)     b=7.6878(4)        c=19.1203(9)
                  alpha=90         beta=94.160(4)     gamma=90
Temperature:      296 K

                Calculated                  Reported
Volume          2597.7(2)                   2597.7(2)
Space group     P 21/n                      P 21/n
Hall group      -P 2yn                      -P 2yn
Moiety formula  C26 H26 N6 O3 S, H2 O       ?
Sum formula     C26 H28 N6 O4 S             C26 H28 N6 O4 S
Mr              520.60                      520.60
Dx,g cm-3       1.331                       1.331
Z               4                           4
Mu (mm-1)       0.169                       0.169
F000            1096.0                      1096.0
F000'           1096.93
h,k,lmax        24,10,26                    23,10,25
Nref            7407                        6550
Tmin,Tmax       0.971,0.990                 0.734,1.000
Tmin'           0.946

Correction method= # Reported T Limits: Tmin=0.734 Tmax=1.000
AbsCorr = GAUSSIAN

Data completeness= 0.884           Theta(max)= 29.748

R(reflections)= 0.0573( 4053)                     wR2(reflections)=
                                                  0.1640( 6550)
S = 1.030                  Npar= 344
```

The following ALERTS were generated. Each ALERT has the format
test-name_ALERT_alert-type_alert-level.
Click on the hyperlinks for more details of the test.

Alert level B

```
PLAT910_ALERT_3_B Missing # of FCF Reflection(s) Below Theta(Min).         11 Note
```

Alert level C

```
PLAT241_ALERT_2_C High   'MainMol' Ueq as Compared to Neighbors of         O3 Check
PLAT906_ALERT_3_C Large K Value in the Analysis of Variance ......      4.499 Check
```

Alert level G

```
PLAT002_ALERT_2_G Number of Distance or Angle Restraints on AtSite          3 Note
PLAT172_ALERT_4_G The CIF-Embedded .res File Contains DFIX Records          2 Report
PLAT793_ALERT_4_G Model has Chirality at C8          (Centro SPGR)          S Verify
PLAT860_ALERT_3_G Number of Least-Squares Restraints .............          3 Note
PLAT883_ALERT_1_G No Info/Value for _atom_sites_solution_primary .     Please Do !
PLAT912_ALERT_4_G Missing # of FCF Reflections Above STh/L=  0.600        797 Note
PLAT941_ALERT_3_G Average HKL Measurement Multiplicity ...........        3.8 Low
PLAT978_ALERT_2_G Number C-C Bonds with Positive Residual Density.          2 Info
```

---

0 **ALERT level A** = Most likely a serious problem - resolve or explain
1 **ALERT level B** = A potentially serious problem, consider carefully
2 **ALERT level C** = Check. Ensure it is not caused by an omission or oversight
8 **ALERT level G** = General information/check it is not something unexpected

1 ALERT type 1 CIF construction/syntax error, inconsistent or missing data
3 ALERT type 2 Indicator that the structure model may be wrong or deficient
4 ALERT type 3 Indicator that the structure quality may be low
3 ALERT type 4 Improvement, methodology, query or suggestion
0 ALERT type 5 Informative message, check

It is advisable to attempt to resolve as many as possible of the alerts in all categories. Often the minor alerts point to easily fixed oversights, errors and omissions in your CIF or refinement strategy, so attention to these fine details can be worthwhile. In order to resolve some of the more serious problems it may be necessary to carry out additional measurements or structure refinements. However, the purpose of your study may justify the reported deviations and the more serious of these should normally be commented upon in the discussion or experimental section of a paper or in the "special_details" fields of the CIF. checkCIF was carefully designed to identify outliers and unusual parameters, but every test has its limitations and alerts that are not important in a particular case may appear. Conversely, the absence of alerts does not guarantee there are no aspects of the results needing attention. It is up to the individual to critically assess their own results and, if necessary, seek expert advice.

Publication of your CIF in IUCr journals

A basic structural check has been run on your CIF. These basic checks will be run on all CIFs submitted for publication in IUCr journals (*Acta Crystallographica*, *Journal of Applied Crystallography*, *Journal of Synchrotron Radiation*); however, if you intend to submit to *Acta Crystallographica Section C* or *E* or *IUCrData*, you should make sure that full publication checks are run on the final version of your CIF prior to submission.

Publication of your CIF in other journals

Please refer to the *Notes for Authors* of the relevant journal for any special instructions relating to CIF submission.

PLATON version of 12/09/2022; check.def file version of 09/08/2022

Datablock bmk1936b - ellipsoid plot

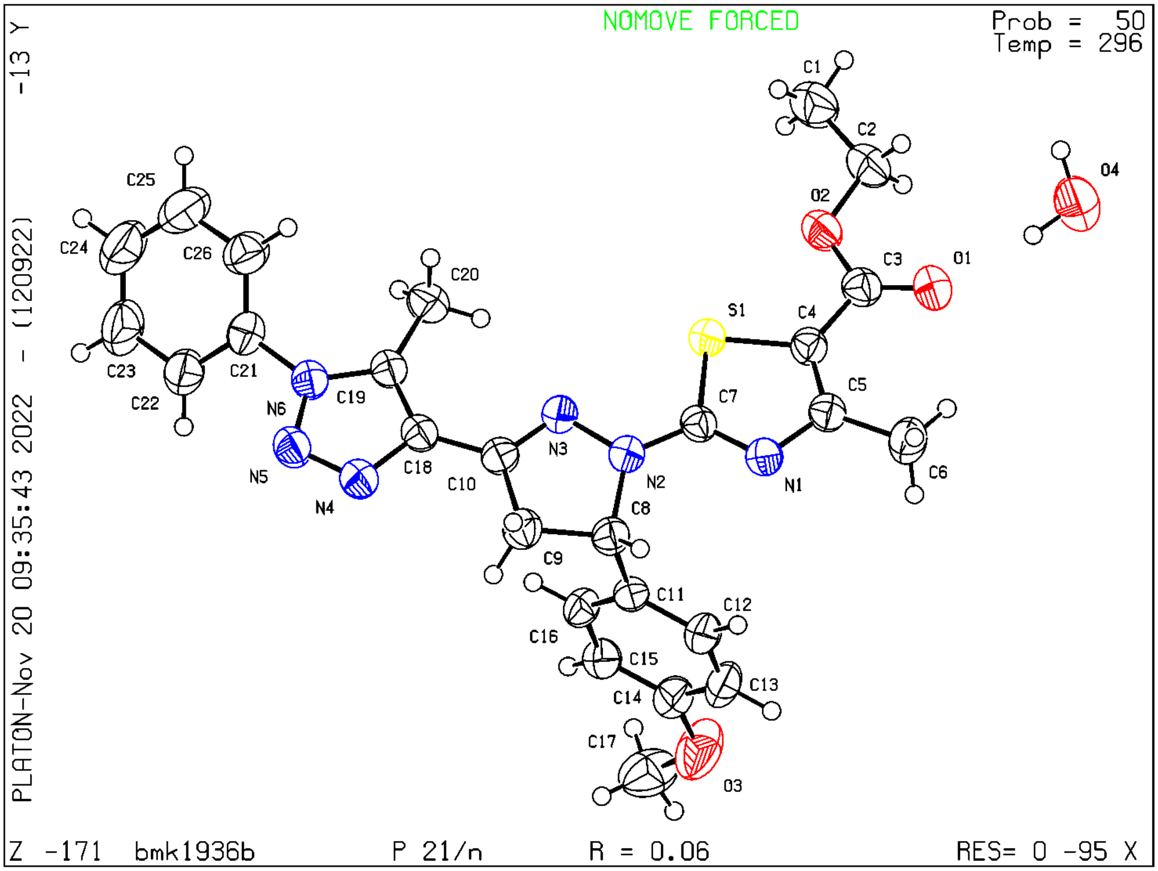

Supplement: Supplementary file 1 [file molecules-27-08904-s001.zip › Supplementary Materials/26 Compound 8 bmk1936b checkcif.pdf]
