# Supplementary material for: Synthesis and Characterization of Novel 2-(1,2,3-Triazol-4-yl)-4,5-dihydro-1H-pyrazol-1-yl)thiazoles and 2-(4,5-Dihydro-1H-pyrazol-1-yl)-4-(1H-1,2,3-triazol-4-yl)thiazoles
Source: Molecules. 2022 Dec 14;27(24):8904. doi: 10.3390/molecules27248904 (PMC9786072; doi:10.3390/molecules27248904)

checkCIF/PLATON report

You have not supplied any structure factors. As a result the full set of tests cannot be run.

THIS REPORT IS FOR GUIDANCE ONLY. IF USED AS PART OF A REVIEW PROCEDURE FOR PUBLICATION, IT SHOULD NOT REPLACE THE EXPERTISE OF AN EXPERIENCED CRYSTALLOGRAPHIC REFEREE.

No syntax errors found. CIF dictionary Interpreting this report

Datablock: bmk1919b

```
Bond precision:   C-C = 0.0036 A          Wavelength=0.71073

Cell:           a=6.5793(4)        b=13.1589(8)      c=16.0368(12)
                alpha=65.959(6)    beta=82.697(5)    gamma=87.701(5)
Temperature:    298 K

                 Calculated                        Reported
Volume           1257.58(16)                       1257.58(15)
Space group      P -1                              P -1
Hall group       -P 1                              -P 1
Moiety formula   C29 H20 F2 N6 O S                 ?
Sum formula      C29 H20 F2 N6 O S                 C29 H20 F2 N6 O S
Mr               538.57                            538.57
Dx,g cm-3        1.422                             1.422
Z                2                                 2
Mu (mm-1)        0.180                             0.180
F000             556.0                             556.0
F000'            556.49
h,k,lmax         9,18,22                           9,18,21
Nref             7248                              5939
Tmin,Tmax        0.970,0.984                       0.655,1.000
Tmin'            0.952

Correction method= # Reported T Limits: Tmin=0.655 Tmax=1.000
AbsCorr = GAUSSIAN

Data completeness= 0.819           Theta(max)= 29.849

R(reflections)= 0.0538( 3972)                         wR2(reflections)=
                                                      0.1386( 5939)
S = 1.031                 Npar= 353
```

The following ALERTS were generated. Each ALERT has the format
test-name_ALERT_alert-type_alert-level.
Click on the hyperlinks for more details of the test.

Alert level C

PLAT334_ALERT_2_C Small <C-C> Benzene Dist. C1 -C6 . 1.37 Ang.
PLAT334_ALERT_2_C Small <C-C> Benzene Dist. C13 -C18 . 1.37 Ang.

Alert level G

PLAT012_ALERT_1_G N.O.K. _shelx_res_checksum Found in CIF ...... Please Check
PLAT398_ALERT_2_G Deviating C-O-C Angle From 120 for O1 . 105.6 Degree
PLAT793_ALERT_4_G Model has Chirality at C12 (Centro SPGR) S Verify
PLAT883_ALERT_1_G No Info/Value for _atom_sites_solution_primary . Please Do !
PLAT941_ALERT_3_G Average HKL Measurement Multiplicity .... 1.9 Low

0 **ALERT level A** = Most likely a serious problem - resolve or explain
0 **ALERT level B** = A potentially serious problem, consider carefully
2 **ALERT level C** = Check. Ensure it is not caused by an omission or oversight
5 **ALERT level G** = General information/check it is not something unexpected

2 ALERT type 1 CIF construction/syntax error, inconsistent or missing data
3 ALERT type 2 Indicator that the structure model may be wrong or deficient
1 ALERT type 3 Indicator that the structure quality may be low
1 ALERT type 4 Improvement, methodology, query or suggestion
0 ALERT type 5 Informative message, check

It is advisable to attempt to resolve as many as possible of the alerts in all categories. Often the minor alerts point to easily fixed oversights, errors and omissions in your CIF or refinement strategy, so attention to these fine details can be worthwhile. In order to resolve some of the more serious problems it may be necessary to carry out additional measurements or structure refinements. However, the purpose of your study may justify the reported deviations and the more serious of these should normally be commented upon in the discussion or experimental section of a paper or in the "special_details" fields of the CIF. checkCIF was carefully designed to identify outliers and unusual parameters, but every test has its limitations and alerts that are not important in a particular case may appear. Conversely, the absence of alerts does not guarantee there are no aspects of the results needing attention. It is up to the individual to critically assess their own results and, if necessary, seek expert advice.

Publication of your CIF in IUCr journals

A basic structural check has been run on your CIF. These basic checks will be run on all CIFs submitted for publication in IUCr journals (*Acta Crystallographica*, *Journal of Applied Crystallography*, *Journal of Synchrotron Radiation*); however, if you intend to submit to *Acta Crystallographica Section C* or *E* or *IUCrData*, you should make sure that full publication checks are run on the final version of your CIF prior to submission.

Publication of your CIF in other journals

Please refer to the *Notes for Authors* of the relevant journal for any special instructions relating to CIF submission.

PLATON version of 12/09/2022; check.def file version of 09/08/2022

Datablock bmk1919b - ellipsoid plot

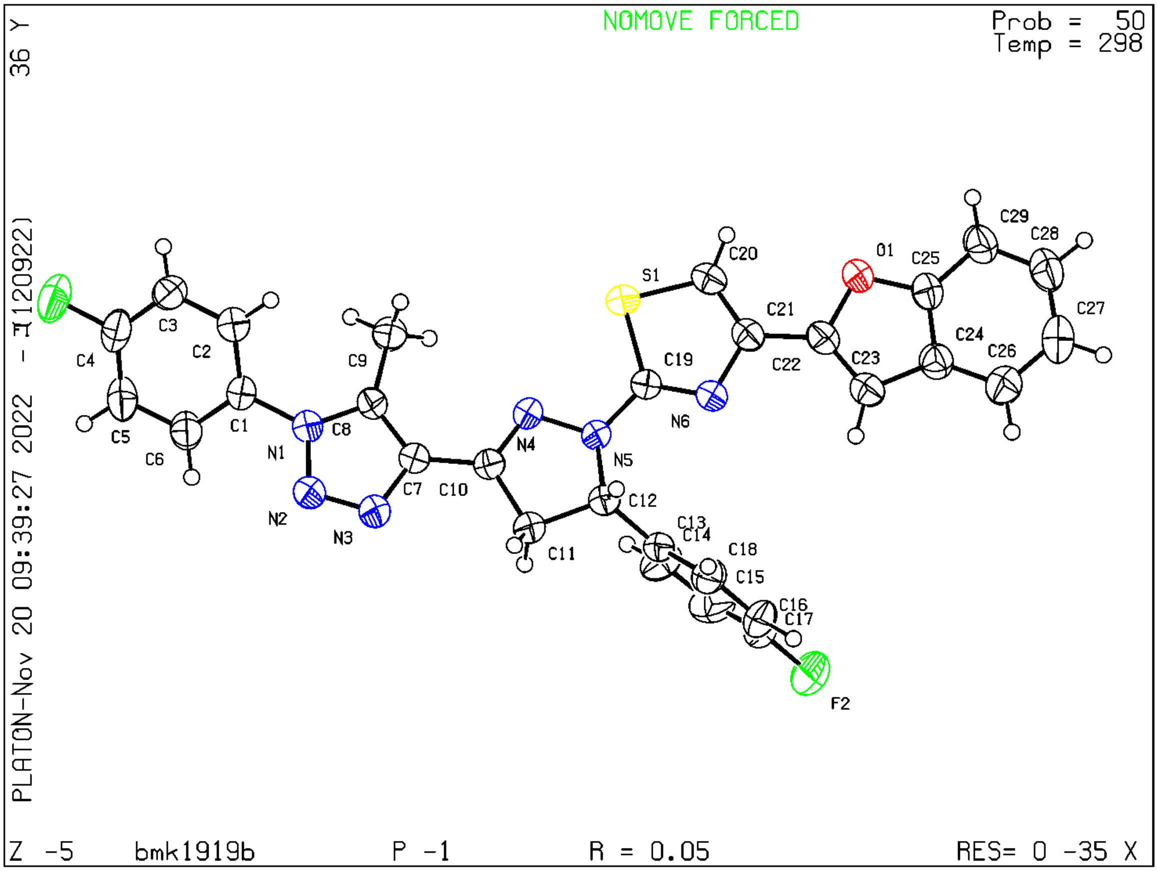

Supplement: Supplementary file 1 [file molecules-27-08904-s001.zip › Supplementary Materials/26 Compound 9 bmk1919b checkcif.pdf]
