# Supplementary material for: Synthesis and Characterization of Novel 2-(1,2,3-Triazol-4-yl)-4,5-dihydro-1H-pyrazol-1-yl)thiazoles and 2-(4,5-Dihydro-1H-pyrazol-1-yl)-4-(1H-1,2,3-triazol-4-yl)thiazoles
Source: Molecules. 2022 Dec 14;27(24):8904. doi: 10.3390/molecules27248904 (PMC9786072; doi:10.3390/molecules27248904)

checkCIF/PLATON report

Structure factors have been supplied for datablock(s) bmk1923b

THIS REPORT IS FOR GUIDANCE ONLY. IF USED AS PART OF A REVIEW PROCEDURE FOR PUBLICATION, IT SHOULD NOT REPLACE THE EXPERTISE OF AN EXPERIENCED CRYSTALLOGRAPHIC REFEREE.

No syntax errors found. CIF dictionary Interpreting this report

Datablock: bmk1923b

Bond precision:	C-C = 0.0036 A	Wavelength=0.71073	
Cell:	a=12.7331(8)	b=21.2977(9)	c=9.7424(5)
	alpha=90	beta=105.329(6)	gamma=90
Temperature:	293 K		

	Calculated	Reported
Volume	2548.0(2)	2548.0(2)
Space group	P 21/c	P 21/c
Hall group	-P 2ybc	-P 2ybc
Moiety formula	C28 H22 Cl F N6 S	?
Sum formula	C28 H22 Cl F N6 S	C28 H22 Cl F N6 S
Mr	529.03	529.02
Dx,g cm-3	1.379	1.379
Z	4	4
Mu (mm-1)	0.269	0.269
F000	1096.0	1096.0
F000'	1097.45	
h,k,lmax	17,29,13	16,27,13
Nref	7258	6378
Tmin,Tmax	0.961,0.968	0.509,1.000
Tmin'	0.928	

Correction method= # Reported T Limits: Tmin=0.509 Tmax=1.000
AbsCorr = GAUSSIAN

Data completeness= 0.879 Theta(max)= 29.720

R(reflections)= 0.0487(4114) wR2(reflections)= 0.1395(6378)

S = 1.036 Npar= 336

The following ALERTS were generated. Each ALERT has the format
test-name_ALERT_alert-type_alert-level.
Click on the hyperlinks for more details of the test.

Alert level C

```
PLAT241_ALERT_2_C High   'MainMol' Ueq as Compared to Neighbors of        C19 Check
PLAT242_ALERT_2_C Low    'MainMol' Ueq as Compared to Neighbors of        C17 Check
PLAT242_ALERT_2_C Low    'MainMol' Ueq as Compared to Neighbors of        C20 Check
PLAT334_ALERT_2_C Small <C-C> Benzene Dist.   C17      -C22      .       1.37 Ang.
PLAT906_ALERT_3_C Large K Value in the Analysis of Variance ......      5.602 Check
PLAT910_ALERT_3_C Missing # of FCF Reflection(s) Below Theta(Min).          9 Note
PLAT934_ALERT_3_C Number of (Iobs-Icalc)/Sigma(W) > 10 Outliers ..          1 Check
```

Alert level G

```
PLAT199_ALERT_1_G Reported _cell_measurement_temperature ..... (K)        293 Check
PLAT200_ALERT_1_G Reported   _diffrn_ambient_temperature ..... (K)        293 Check
PLAT434_ALERT_2_G Short Inter HL..HL Contact Cl1      ..F1       .       2.99 Ang.
                                            -x,-1/2+y,5/2-z  =      2_547 Check
PLAT793_ALERT_4_G Model has Chirality at C14         (Centro SPGR)          R Verify
PLAT883_ALERT_1_G No Info/Value for _atom_sites_solution_primary .     Please Do !
PLAT912_ALERT_4_G Missing # of FCF Reflections Above STh/L=  0.600        759 Note
PLAT941_ALERT_3_G Average HKL Measurement Multiplicity ..........         3.9 Low
PLAT951_ALERT_5_G Calculated (ThMax) and CIF-Reported Kmax Differ           2 Units
PLAT957_ALERT_1_G Calculated (ThMax) and Actual (FCF) Kmax Differ           2 Units
PLAT978_ALERT_2_G Number C-C Bonds with Positive Residual Density.          3 Info
```

---

0 **ALERT level A** = Most likely a serious problem - resolve or explain
0 **ALERT level B** = A potentially serious problem, consider carefully
7 **ALERT level C** = Check. Ensure it is not caused by an omission or oversight
10 **ALERT level G** = General information/check it is not something unexpected

4 ALERT type 1 CIF construction/syntax error, inconsistent or missing data
6 ALERT type 2 Indicator that the structure model may be wrong or deficient
4 ALERT type 3 Indicator that the structure quality may be low
2 ALERT type 4 Improvement, methodology, query or suggestion
1 ALERT type 5 Informative message, check

It is advisable to attempt to resolve as many as possible of the alerts in all categories. Often the minor alerts point to easily fixed oversights, errors and omissions in your CIF or refinement strategy, so attention to these fine details can be worthwhile. In order to resolve some of the more serious problems it may be necessary to carry out additional measurements or structure refinements. However, the purpose of your study may justify the reported deviations and the more serious of these should normally be commented upon in the discussion or experimental section of a paper or in the "special_details" fields of the CIF. checkCIF was carefully designed to identify outliers and unusual parameters, but every test has its limitations and alerts that are not important in a particular case may appear. Conversely, the absence of alerts does not guarantee there are no aspects of the results needing attention. It is up to the individual to critically assess their own results and, if necessary, seek expert advice.

Publication of your CIF in IUCr journals

A basic structural check has been run on your CIF. These basic checks will be run on all CIFs submitted for publication in IUCr journals (*Acta Crystallographica*, *Journal of Applied Crystallography*, *Journal of Synchrotron Radiation*); however, if you intend to submit to *Acta Crystallographica Section C* or *E* or *IUCrData*, you should make sure that full publication checks are run on the final version of your CIF prior to submission.

Publication of your CIF in other journals

Please refer to the *Notes for Authors* of the relevant journal for any special instructions relating to CIF submission.

PLATON version of 12/09/2022; check.def file version of 09/08/2022

Datablock bmk1923b - ellipsoid plot

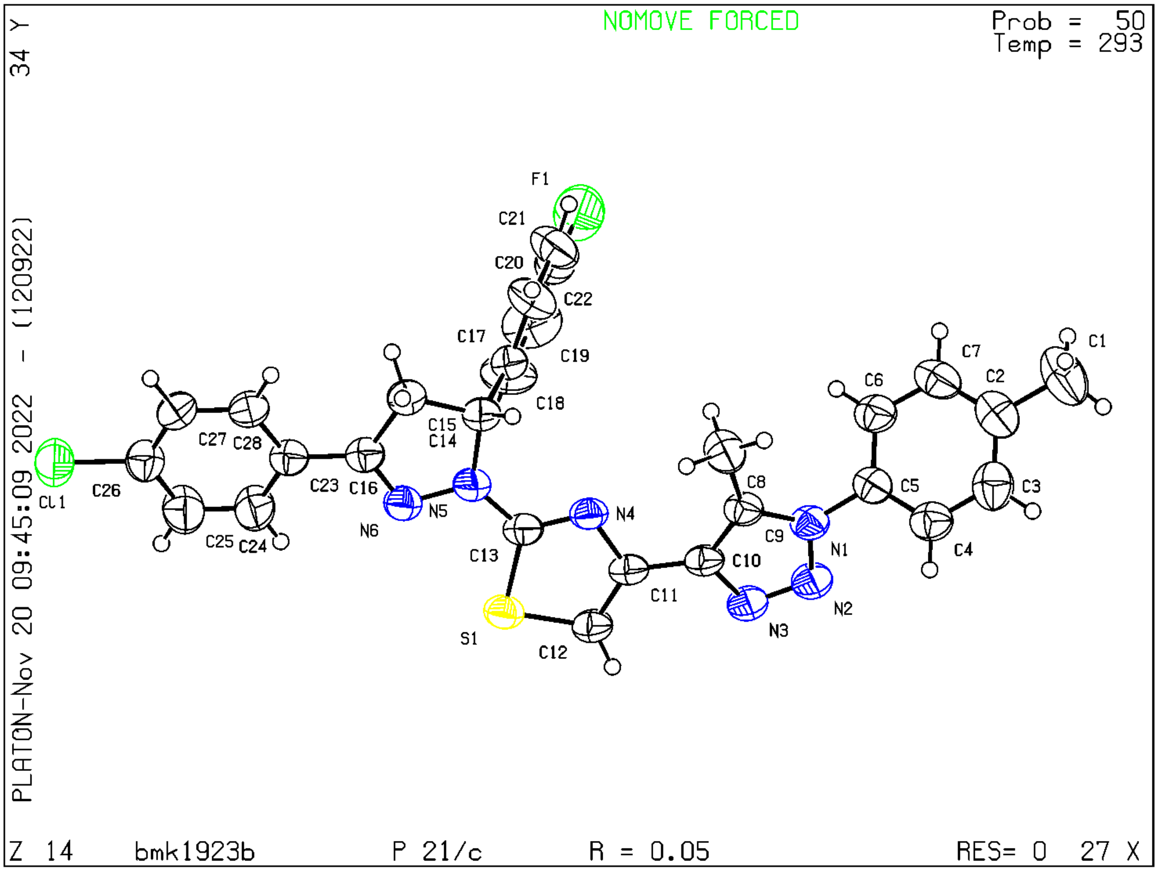

Supplement: Supplementary file 1 [file molecules-27-08904-s001.zip › Supplementary Materials/28 Compound 13 bmk1923b checkcif.pdf]
