# Supplementary material for: Synthesis and Characterization of Novel 2-(1,2,3-Triazol-4-yl)-4,5-dihydro-1H-pyrazol-1-yl)thiazoles and 2-(4,5-Dihydro-1H-pyrazol-1-yl)-4-(1H-1,2,3-triazol-4-yl)thiazoles
Source: Molecules. 2022 Dec 14;27(24):8904. doi: 10.3390/molecules27248904 (PMC9786072; doi:10.3390/molecules27248904)
Supplement: Supplementary file 1 [file molecules-27-08904-s001.zip › Supplementary Materials/30 IR of Compound 4a.pdf]

SHIMADZU

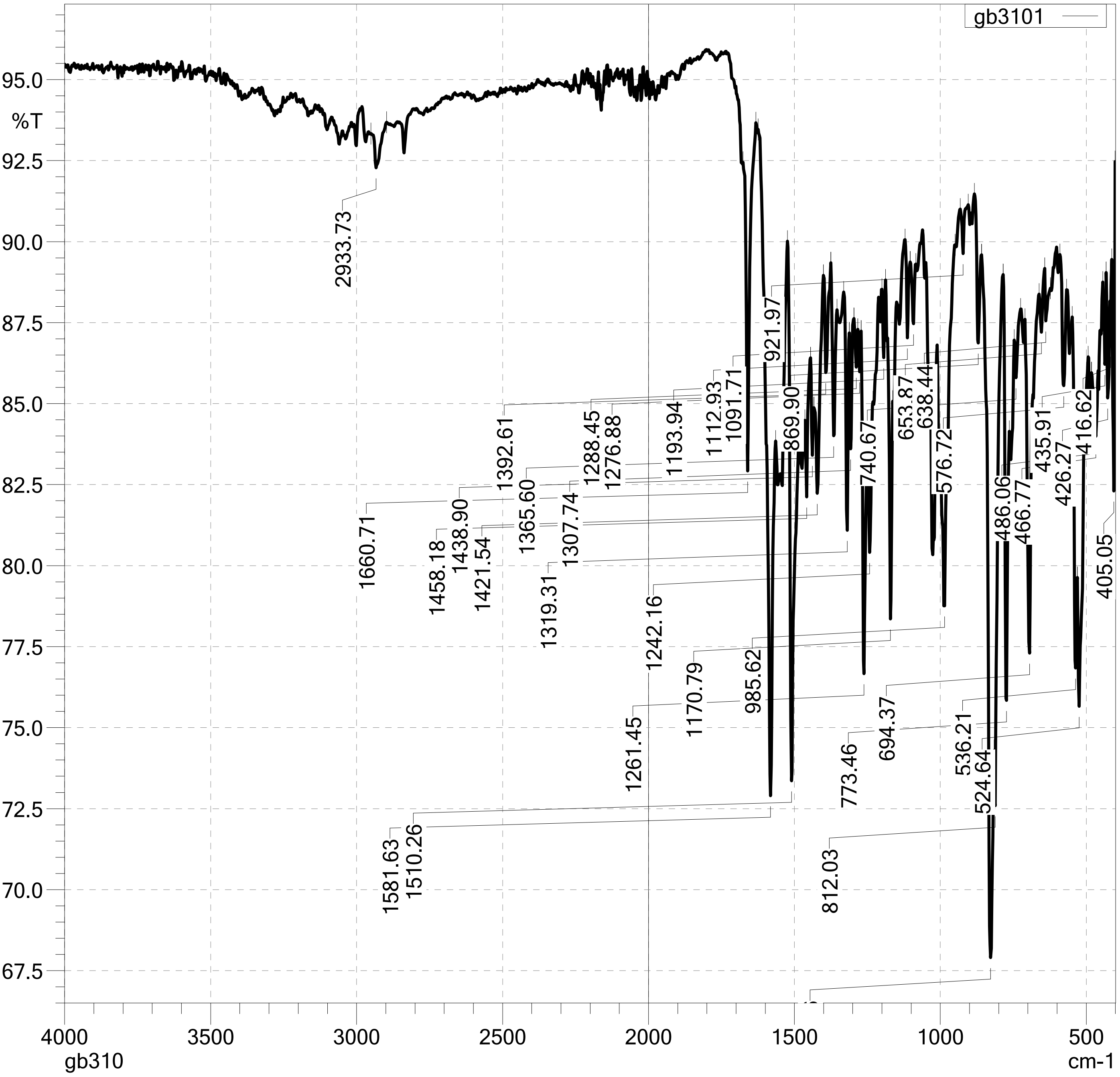

C:¥Users¥Shimadzu¥Desktop¥BK¥071222¥gb3101.ispd

gb310

	Item	Value
2	Sample name	IH_006
3	Sample ID	IH_006
4	Option	IH_006
5	Intensity Mode	%Transmittance
6	Apodization	Happ-Genzel
9	No. of Scans	16

10	Resolution	4 cm-1
