# Supplementary material for: Synthesis and Characterization of Novel 2-(1,2,3-Triazol-4-yl)-4,5-dihydro-1H-pyrazol-1-yl)thiazoles and 2-(4,5-Dihydro-1H-pyrazol-1-yl)-4-(1H-1,2,3-triazol-4-yl)thiazoles
Source: Molecules. 2022 Dec 14;27(24):8904. doi: 10.3390/molecules27248904 (PMC9786072; doi:10.3390/molecules27248904)
Supplement: Supplementary file 1 [file molecules-27-08904-s001.zip › Supplementary Materials/31 IR of Compound 8.pdf]

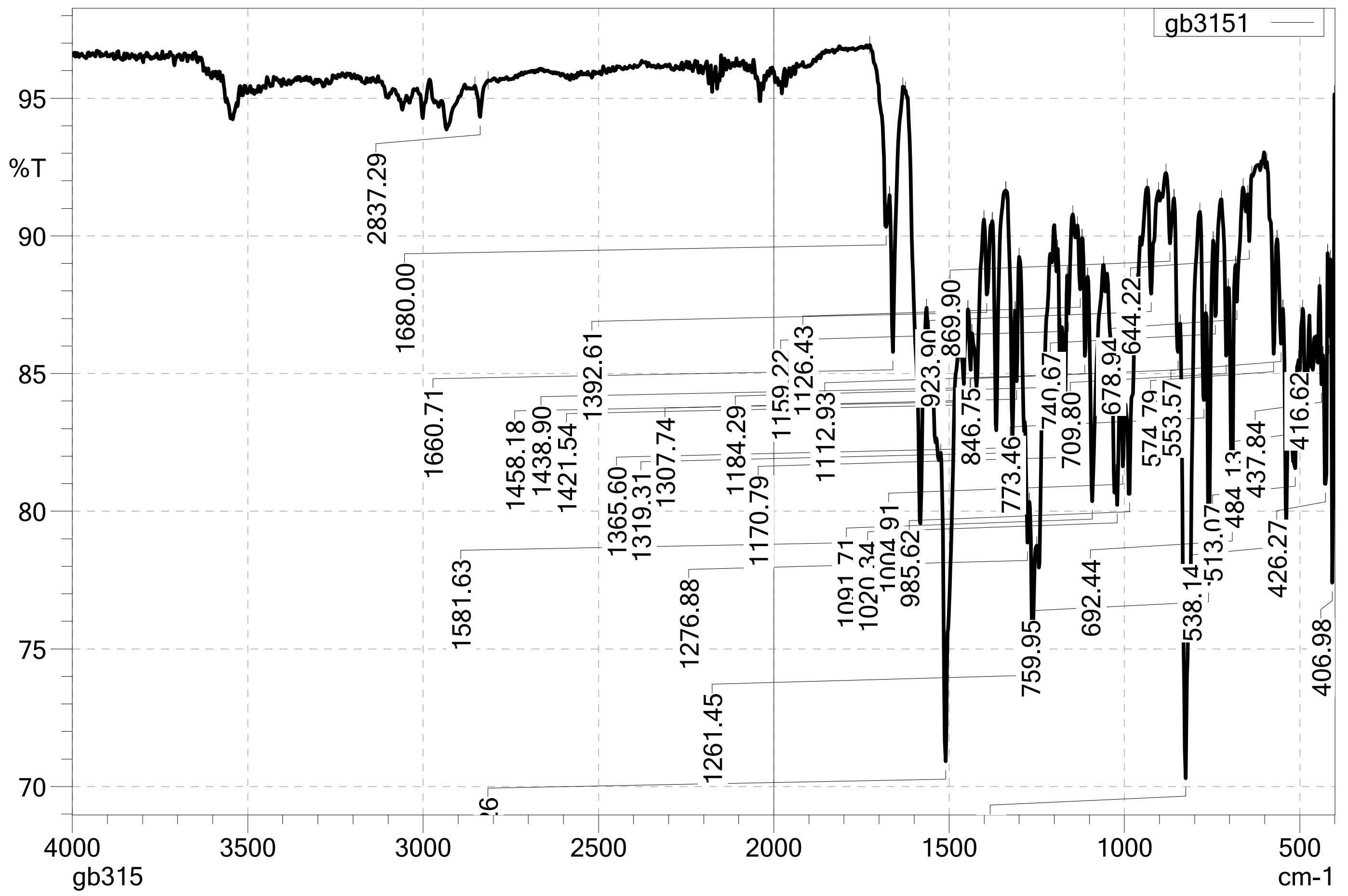

C:¥Users¥Shimadzu¥Desktop¥BK¥071222¥gb3151.ispd
gb315

	Item	Value
2	Sample name	IH_006
3	Sample ID	IH_006
4	Option	IH_006
5	Intensity Mode	%Transmittance
6	Apodization	Happ-Genzel
9	No. of Scans	16
10	Resolution	4 cm-1

No.	Peak	Intensity	Corr. Intensity	Base (H)	Base (L)	Area	Corr. Area	Comment
1	406.98	77.41	14.74	412.77	401.19	180.638	89.719	
2	416.62	86.36	2.90	420.48	412.77	96.391	13.501	
3	426.27	80.99	6.79	433.98	420.48	221.119	52.504	
4	437.84	84.61	2.06	443.63	433.98	135.622	9.467	
5	484.13	83.50	2.45	491.85	480.28	168.480	9.937	
6	513.07	81.56	3.54	528.50	503.42	425.358	48.719	
7	538.14	79.39	6.53	547.78	528.50	320.674	48.971	
8	553.57	86.09	2.11	565.14	547.78	212.256	14.771	
9	574.79	85.72	5.10	594.08	565.14	304.050	52.020	
10	644.22	89.81	1.94	648.08	636.51	103.880	9.722	
11	678.94	87.62	1.84	682.80	661.58	220.278	15.551	
12	692.44	79.57	9.01	704.02	682.80	312.924	69.649	
13	709.80	85.65	3.43	723.31	704.02	221.605	23.350	

14	740.67	87.09	3.11	746.45	723.31	240.625	22.491	
15	759.95	77.36	10.80	767.67	746.45	326.681	82.944	
16	773.46	84.03	4.39	785.03	767.67	222.372	32.043	
17	825.53	70.30	10.42	840.96	815.89	583.831	128.921	
18	846.75	85.79	2.54	858.32	840.96	214.880	25.462	
19	869.90	89.73	2.09	881.47	858.32	209.053	19.854	
20	923.90	87.90	3.82	935.48	902.69	320.890	48.513	
21	985.62	80.63	4.91	997.20	966.34	493.314	64.290	
22	1004.91	81.63	3.00	1010.70	997.20	225.251	16.503	
23	1020.34	80.23	2.24	1024.20	1010.70	237.257	11.657	
24	1091.71	80.36	8.28	1105.21	1058.92	670.989	149.343	
25	1112.93	85.63	3.58	1120.64	1105.21	188.774	22.390	
26	1126.43	88.05	2.06	1134.14	1120.64	146.309	13.290	
27	1159.22	87.19	1.93	1163.08	1147.65	169.574	10.221	
28	1170.79	82.61	5.12	1180.44	1163.08	250.331	35.453	
29	1184.29	85.16	2.66	1190.08	1180.44	126.678	11.957	
30	1261.45	74.69	4.93	1271.09	1249.87	473.359	39.335	
31	1276.88	78.86	2.72	1284.59	1271.09	262.206	16.374	
32	1307.74	84.72	3.22	1311.59	1300.02	152.553	16.702	
33	1319.31	82.77	5.76	1338.60	1311.59	329.237	44.785	
34	1365.60	82.94	7.93	1377.17	1338.60	434.569	90.878	
35	1392.61	87.87	2.70	1400.32	1377.17	245.486	27.094	
36	1421.54	84.52	3.55	1435.04	1400.32	445.529	47.308	
37	1438.90	85.13	1.62	1446.61	1435.04	158.770	7.178	
38	1458.18	84.62	1.92	1469.76	1446.61	327.709	16.160	
39	1510.26	70.92	12.19	1523.76	1475.54	1015.739	238.873	
40	1581.63	79.56	10.11	1624.06	1564.27	752.568	233.960	
41	1660.71	85.79	6.67	1670.35	1631.78	333.546	80.757	
42	1680.00	90.32	2.09	1726.29	1670.35	320.630	-3.615	
43	2837.29	94.32	1.21	2852.72	2814.14	186.930	15.320	
