# Supplementary figures and images for: Synthesis and Characterization of Novel 2-(1,2,3-Triazol-4-yl)-4,5-dihydro-1H-pyrazol-1-yl)thiazoles and 2-(4,5-Dihydro-1H-pyrazol-1-yl)-4-(1H-1,2,3-triazol-4-yl)thiazoles
Source: Molecules. 2022 Dec 14;27(24):8904. doi: 10.3390/molecules27248904 (PMC9786072; doi:10.3390/molecules27248904)

X : parts per Million : 1H

7.8458  
7.8058  
7.7638  
7.7467  
7.6361  
7.6284  
7.6208  
6.9916

3.7921  
3.4098  
3.3087

2.5594  
2.4679

1.0235

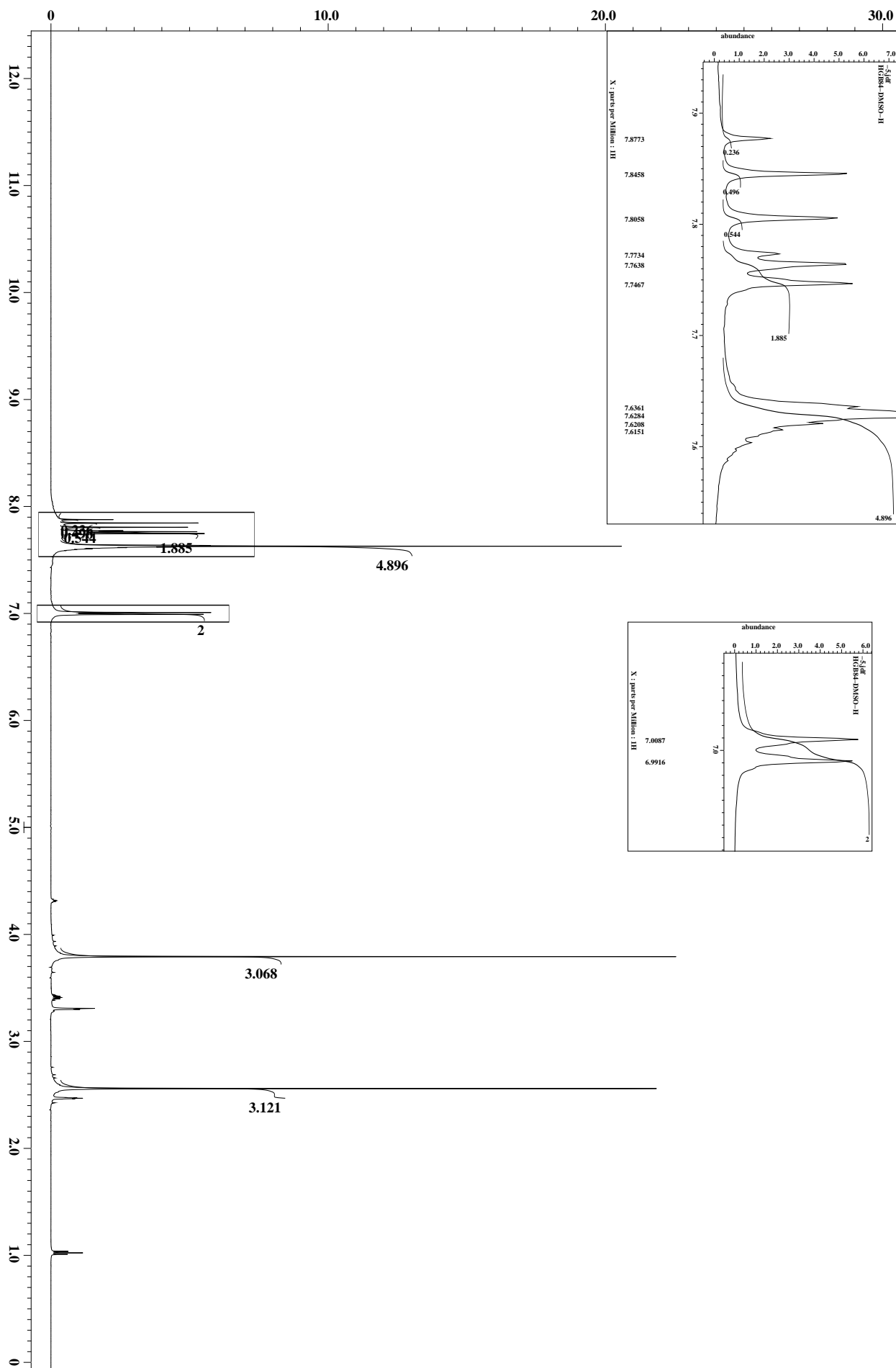

-51df  
HGB84-DMSO-H

Supplement: Supplementary file 1 [file molecules-27-08904-s001.zip › Supplementary Materials/01 H NMR of 3a.pdf]

HGB84-DMSO-C-3.jdf  
HGB84-DMSO-C

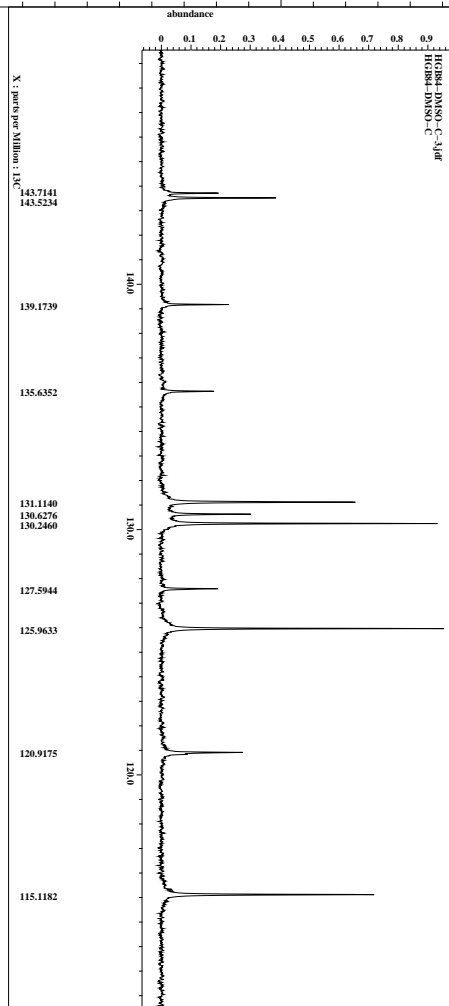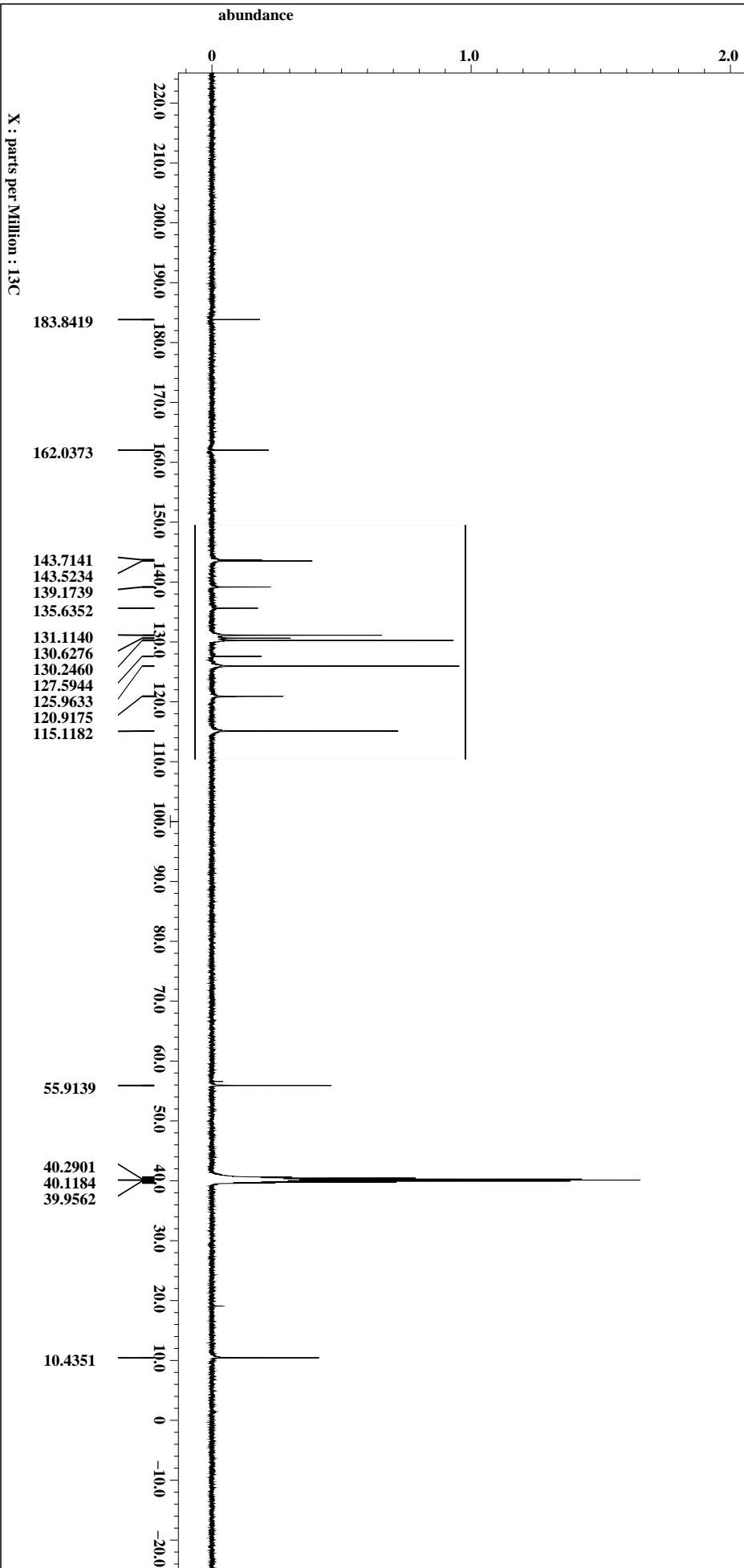

Supplement: Supplementary file 1 [file molecules-27-08904-s001.zip › Supplementary Materials/02 C NMR of 3a.pdf]

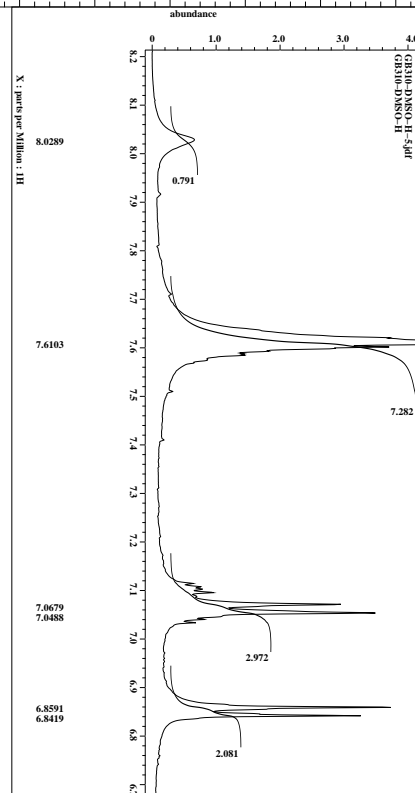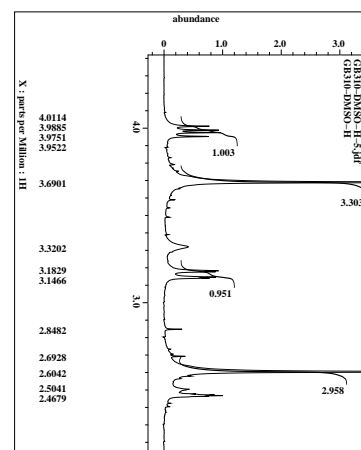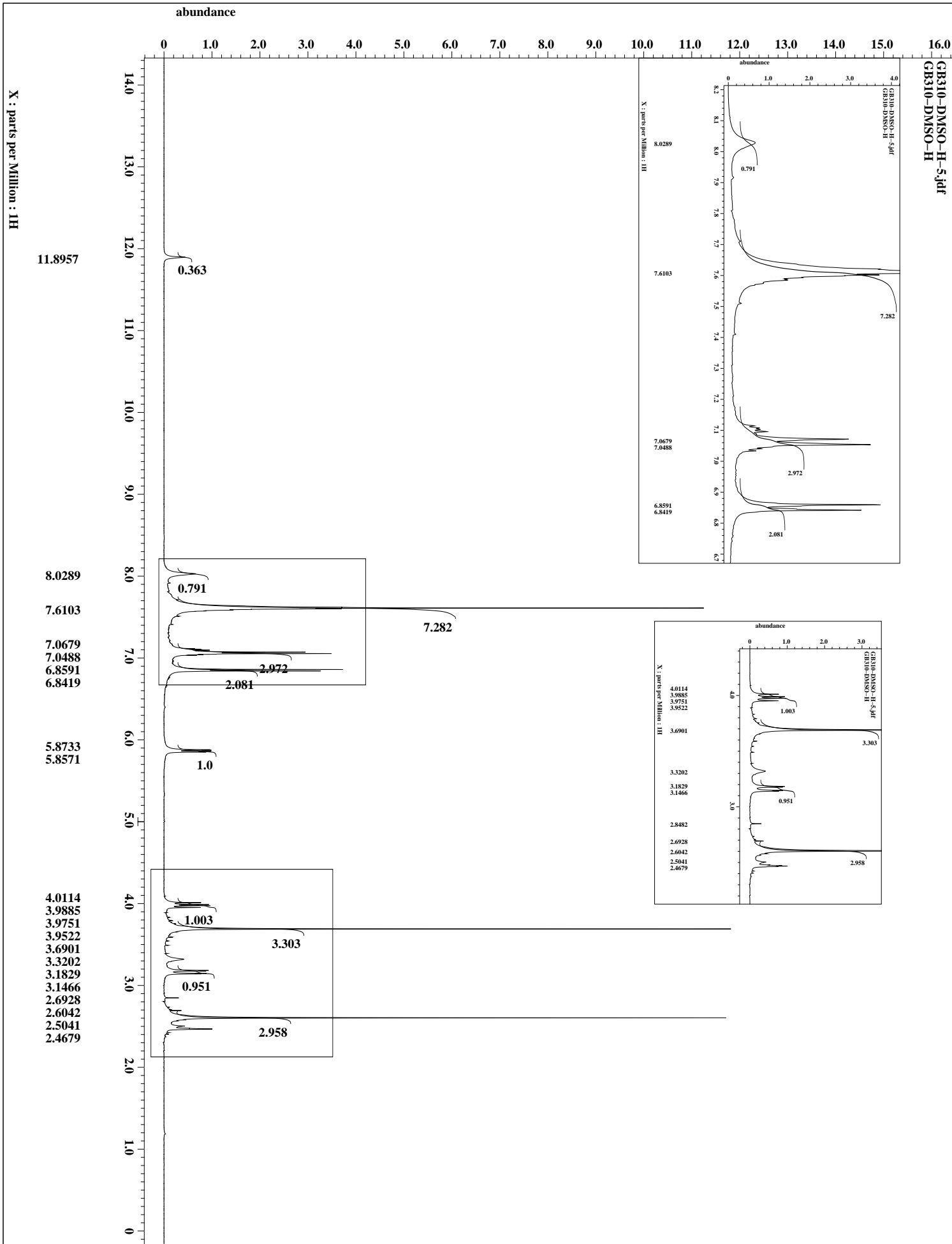

Supplement: Supplementary file 1 [file molecules-27-08904-s001.zip › Supplementary Materials/03 H NMR of 4a.pdf]

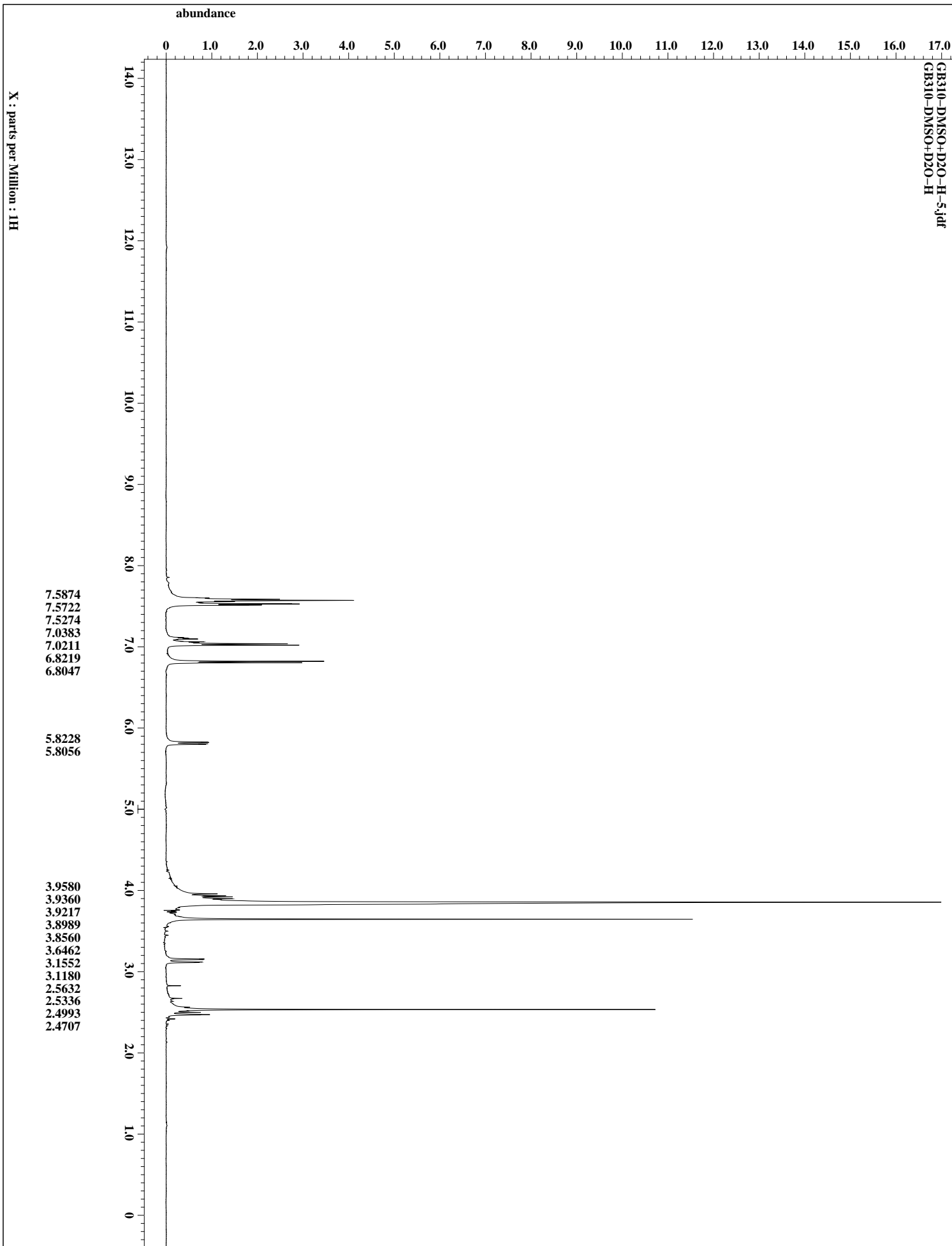

Supplement: Supplementary file 1 [file molecules-27-08904-s001.zip › Supplementary Materials/04 H NMR of 4a D2O.pdf]

GB310-DMSO-13C-3.jdf  
GB310-DMSO-13C

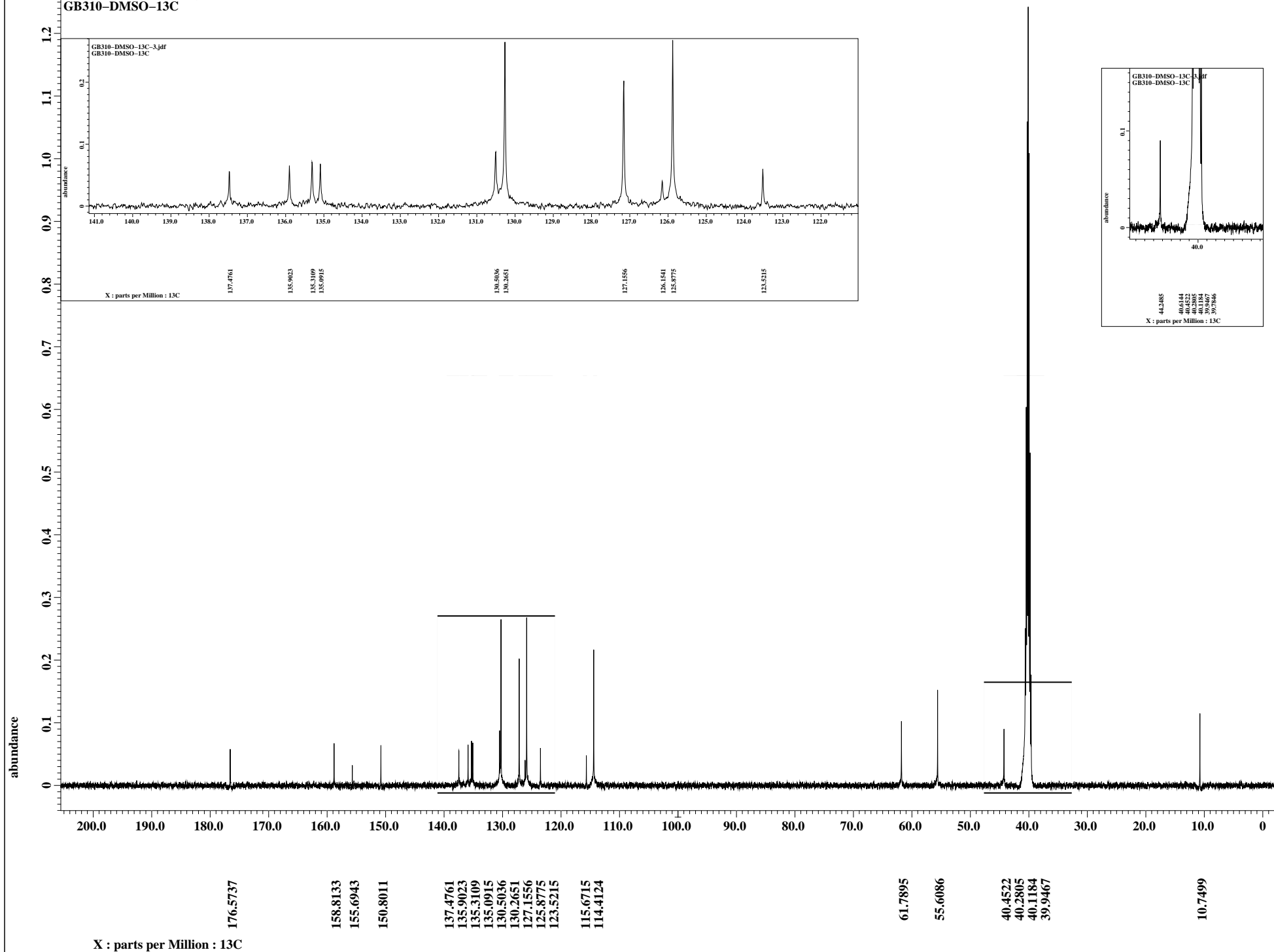

Supplement: Supplementary file 1 [file molecules-27-08904-s001.zip › Supplementary Materials/05 C NMR of 4a.pdf]

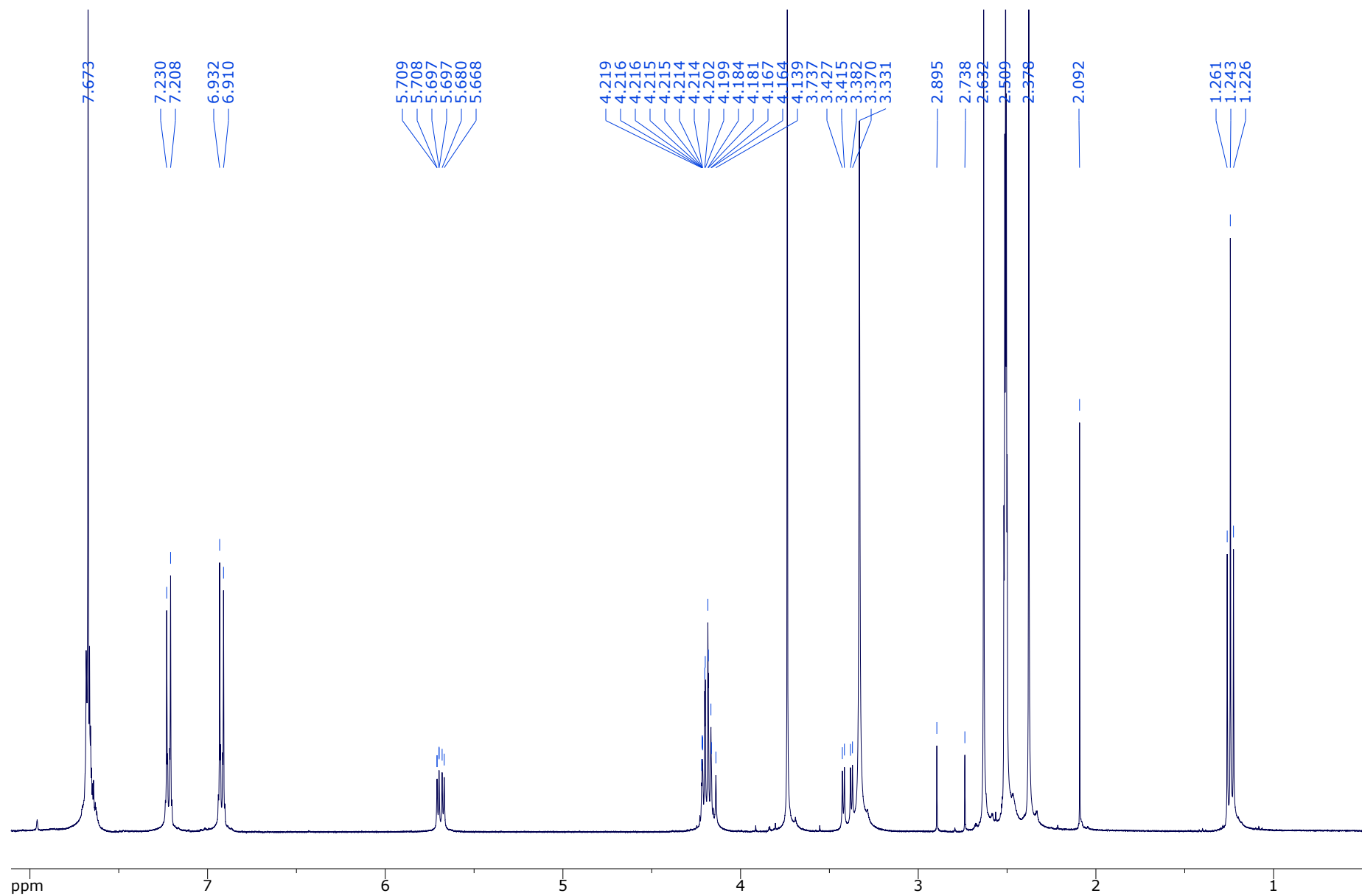

**Fig. S6.**  $^1\text{H}$ -NMR spectrum of compound **8** in DMSO- $d_6$

Supplement: Supplementary file 1 [file molecules-27-08904-s001.zip › Supplementary Materials/06 H NMR of 8.pdf]

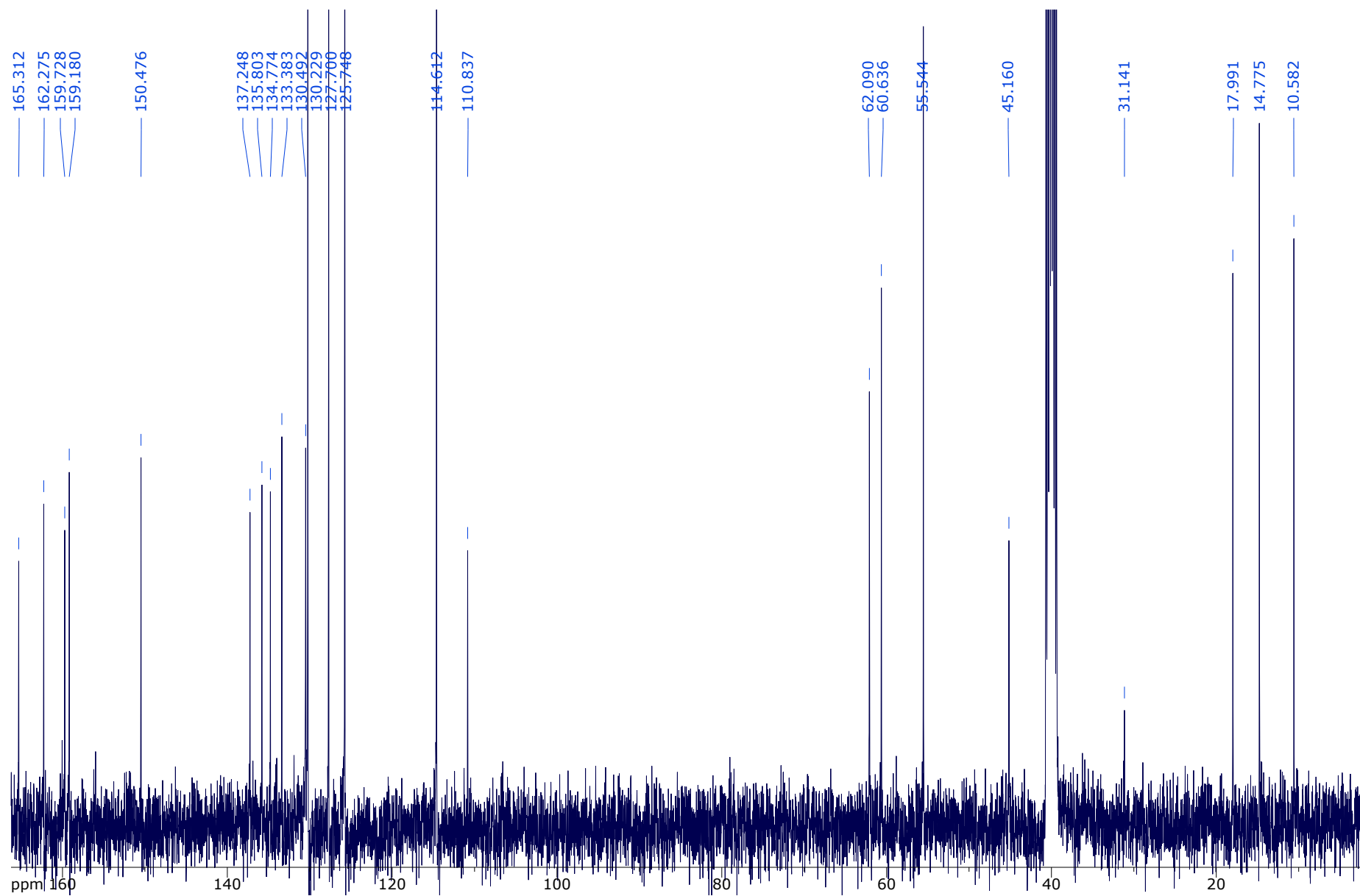

**Fig. S7.** <sup>13</sup>C-NMR spectrum of compound **8** in DMSO-d<sub>6</sub>

Supplement: Supplementary file 1 [file molecules-27-08904-s001.zip › Supplementary Materials/07 C NMR of 8.pdf]

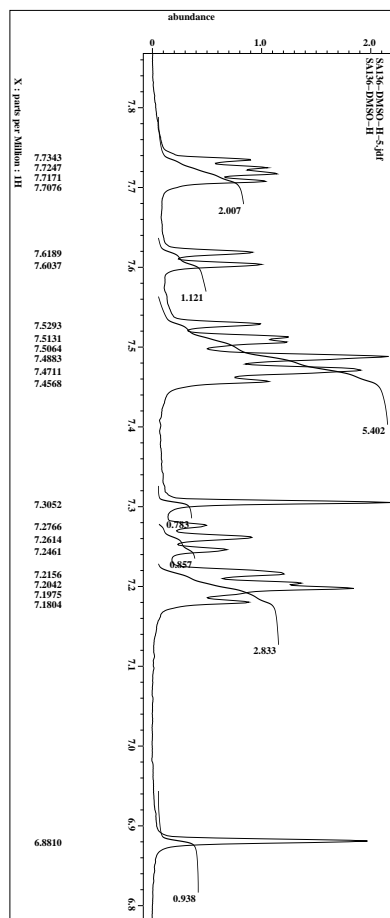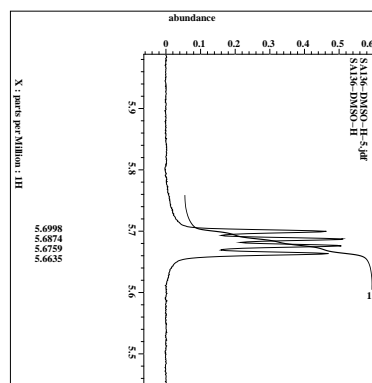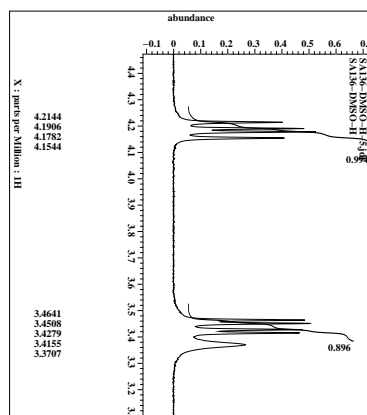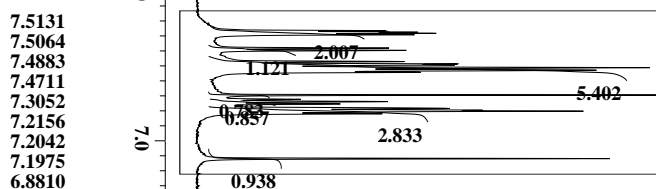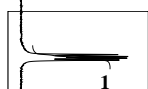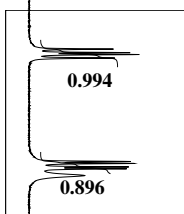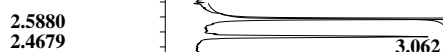

Supplement: Supplementary file 1 [file molecules-27-08904-s001.zip › Supplementary Materials/08 H NMR of 9.pdf]

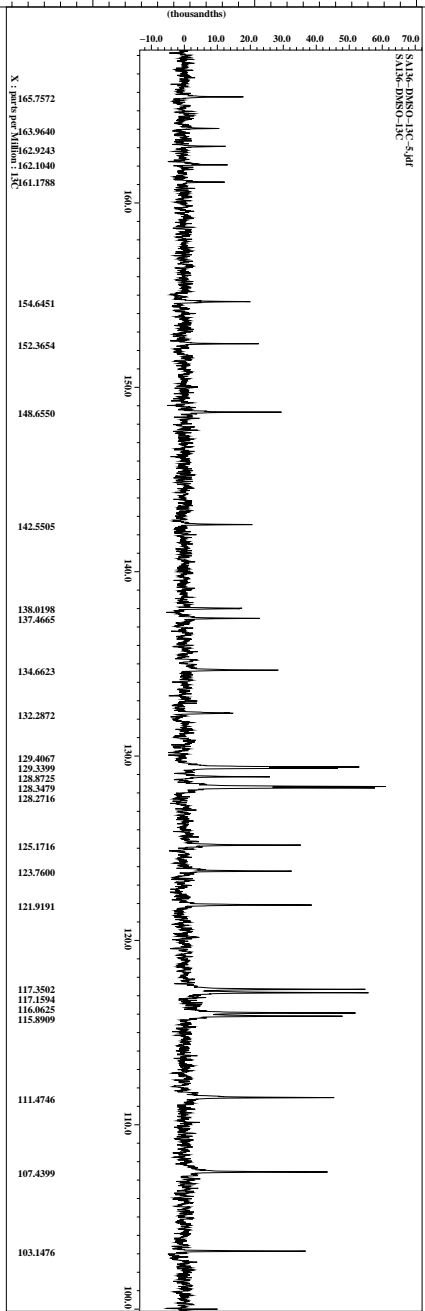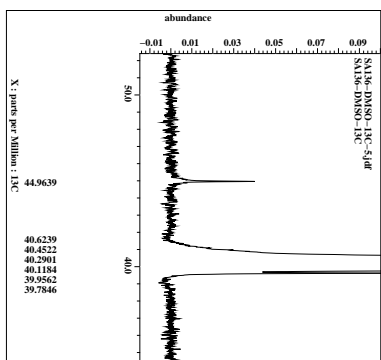

X : parts per Million : 13C

165.7572  
163.9640  
162.9243  
162.1040  
154.6451  
152.3654  
148.6550  
142.5505  
137.4665  
134.6623  
129.4067  
129.3399  
128.8725  
128.3479  
128.2716  
125.1716  
123.7600  
121.9191  
117.3502  
117.1594  
116.0625  
115.8909  
111.4746  
107.4399  
103.1476

63.0008

40.4522  
40.2901  
40.1184  
39.9562

10.4828

Supplement: Supplementary file 1 [file molecules-27-08904-s001.zip › Supplementary Materials/09 C NMR of 9.pdf]

SA138-DMSO-1H-5.jdf  
SA138-DMSO-1H

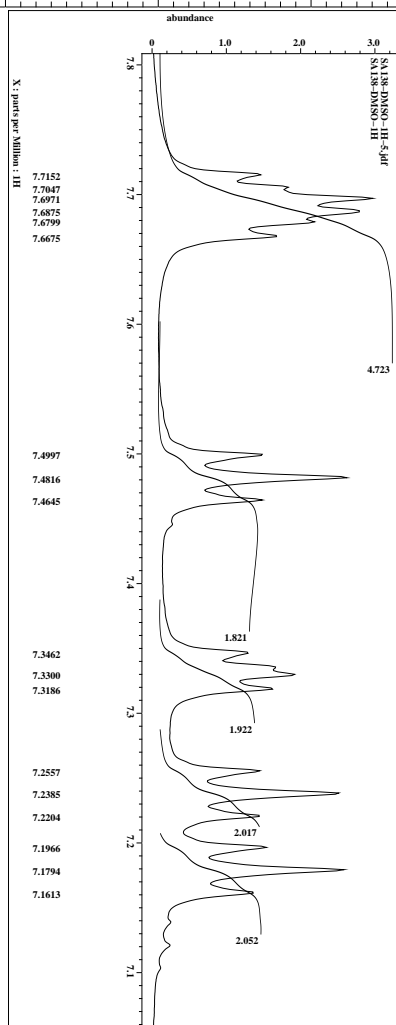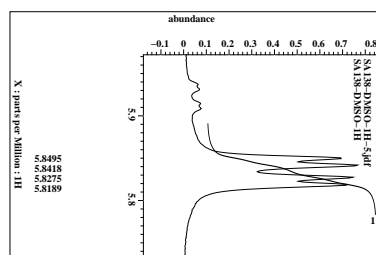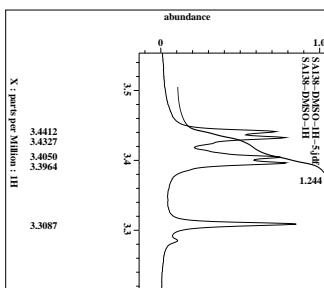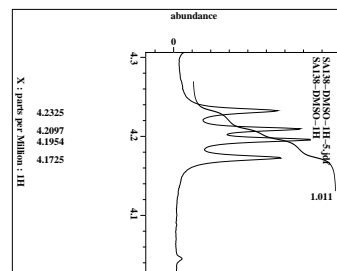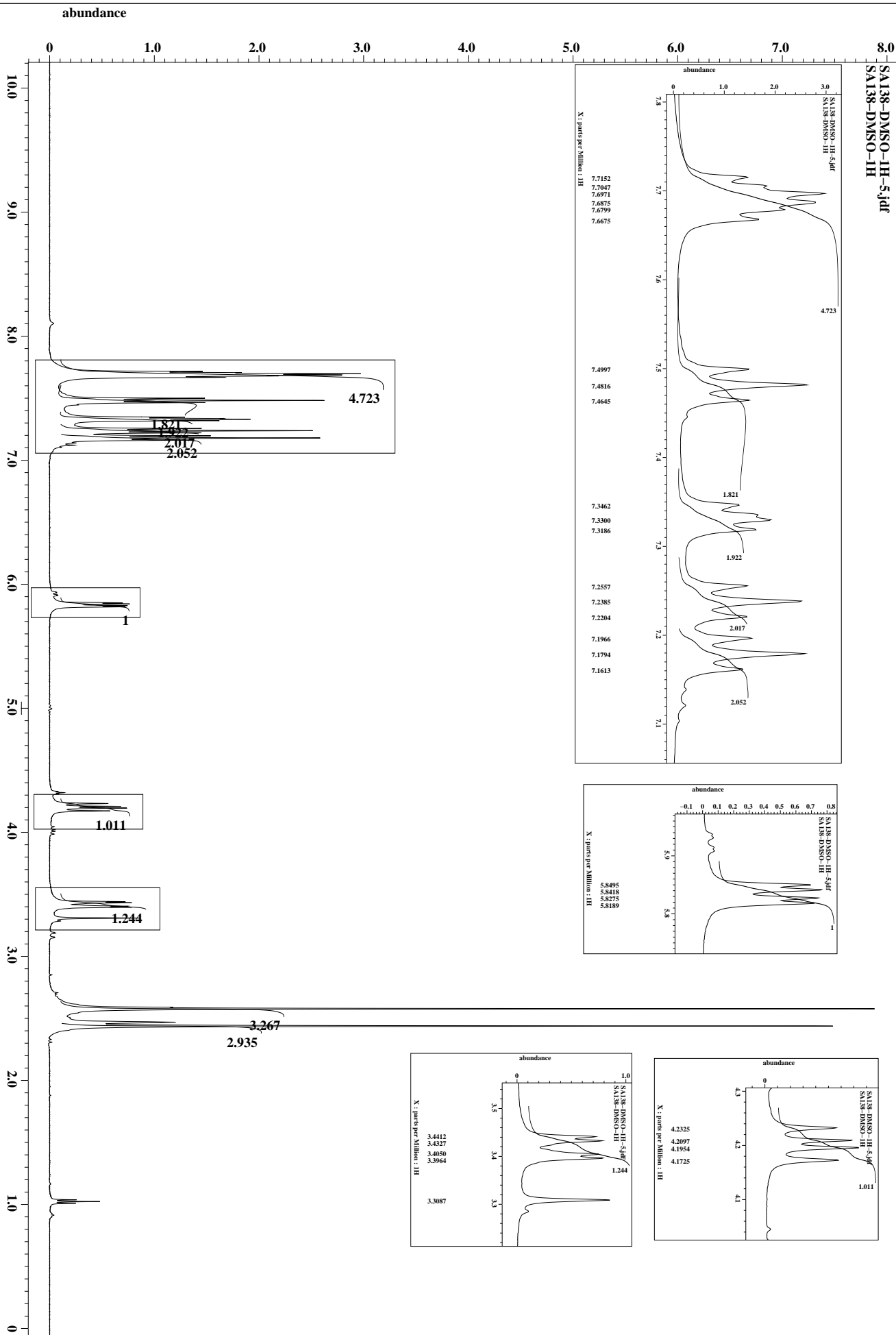

X : parts per Million : 1H

Supplement: Supplementary file 1 [file molecules-27-08904-s001.zip › Supplementary Materials/10 H NMR of 10.pdf]

SA138-DMSO-13C-4.jdf  
SA138-DMSO-13C

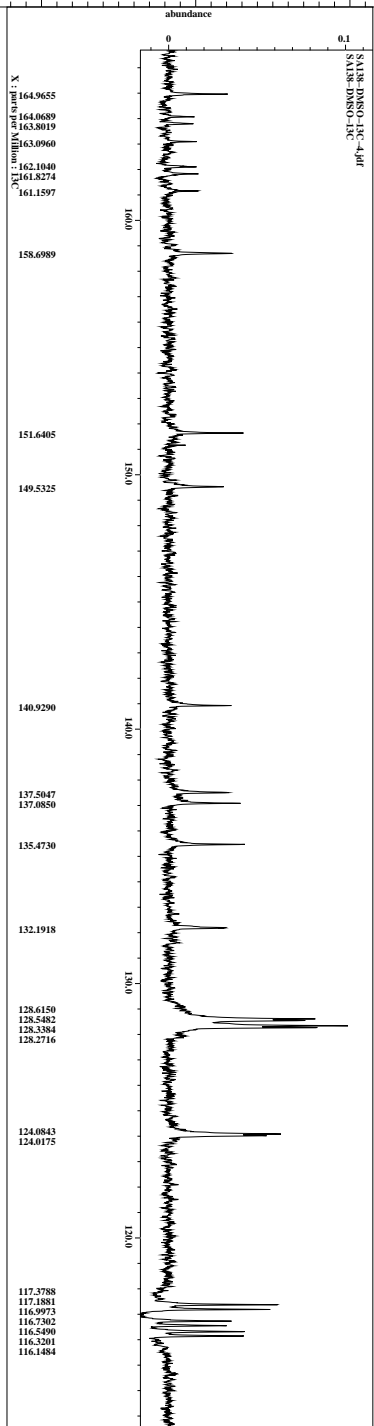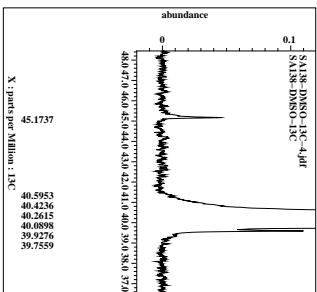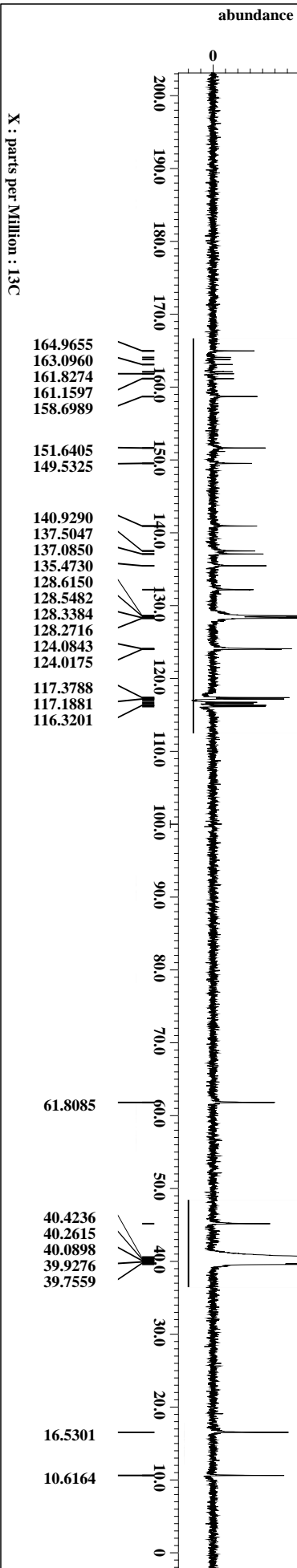

Supplement: Supplementary file 1 [file molecules-27-08904-s001.zip › Supplementary Materials/11 C NMR of 10.pdf]

SA357-DMSO-1H-5Jdf  
SA357-DMSO-1H

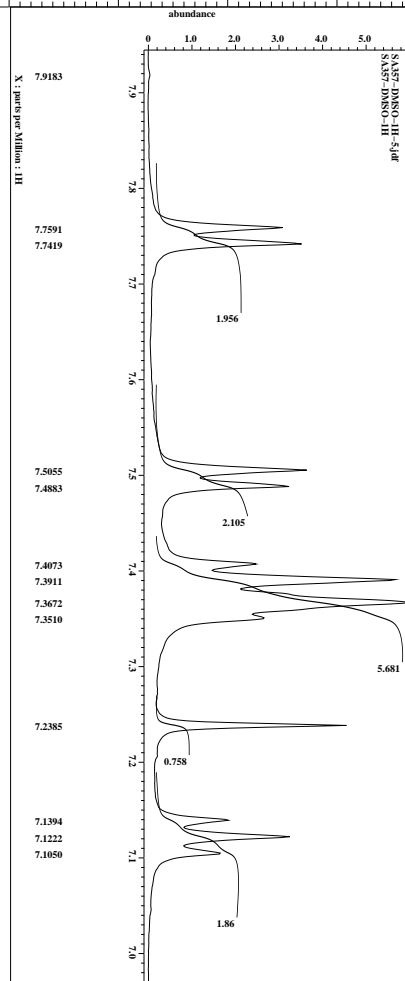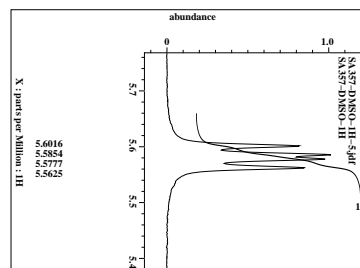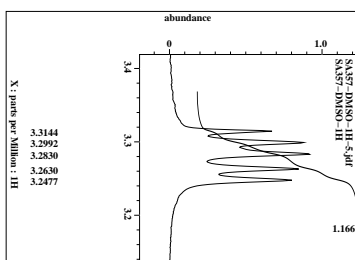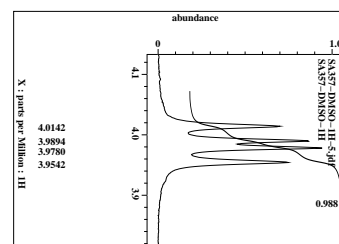

7.7591  
7.7419  
7.5055  
7.4883  
7.3911  
7.3672  
7.3510  
7.2385  
7.1222

5.5854  
5.5777  
5.5625

3.9894  
3.9780  
3.9542

3.2992  
3.2830  
3.2630

2.4688  
2.3697  
2.1466

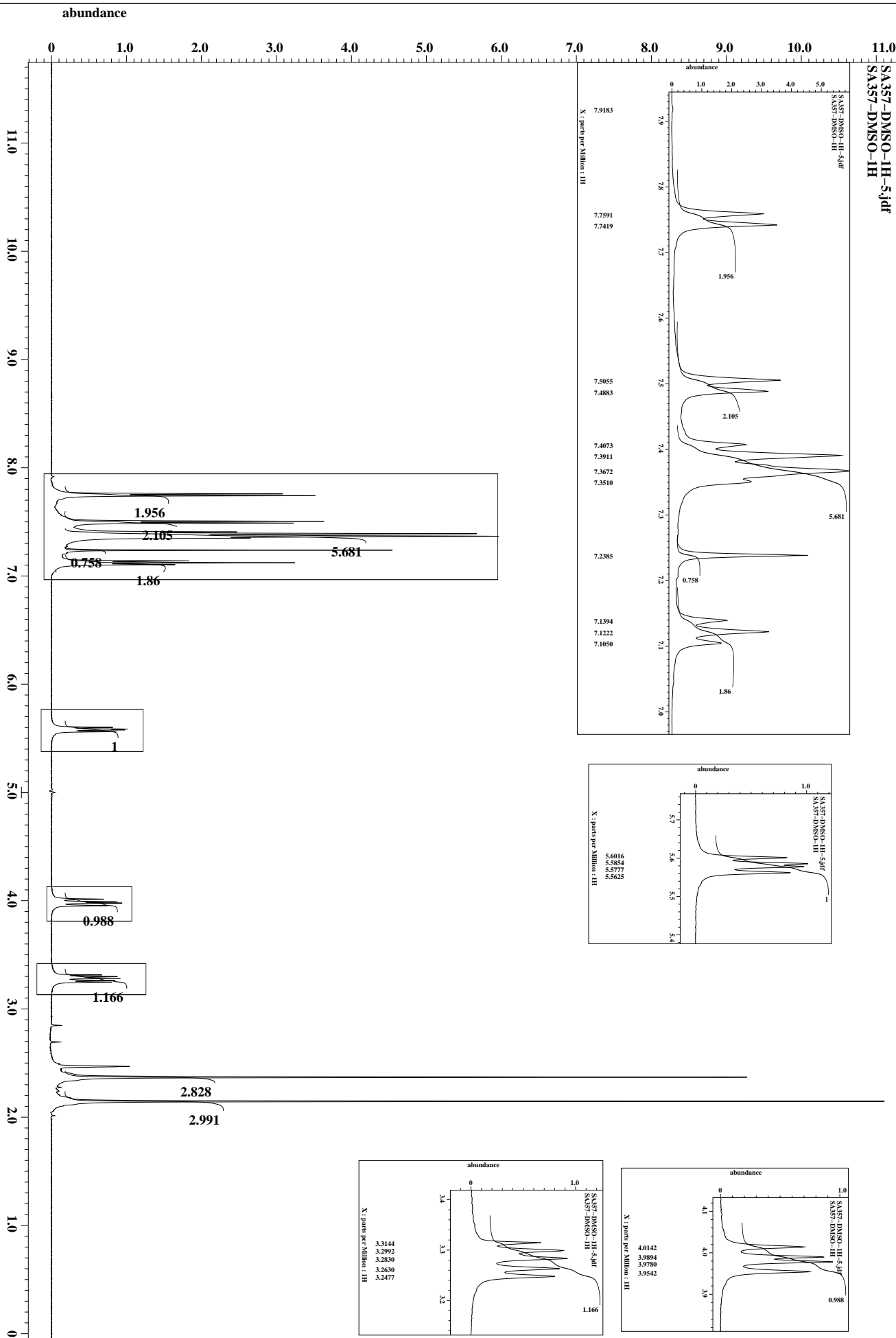

X : parts per Million : 1H

Supplement: Supplementary file 1 [file molecules-27-08904-s001.zip › Supplementary Materials/12 H NMR of 13.pdf]

SA357-DMSO-13C-4.jdf  
M.BEKHEIT/SA357-DMSO-13C

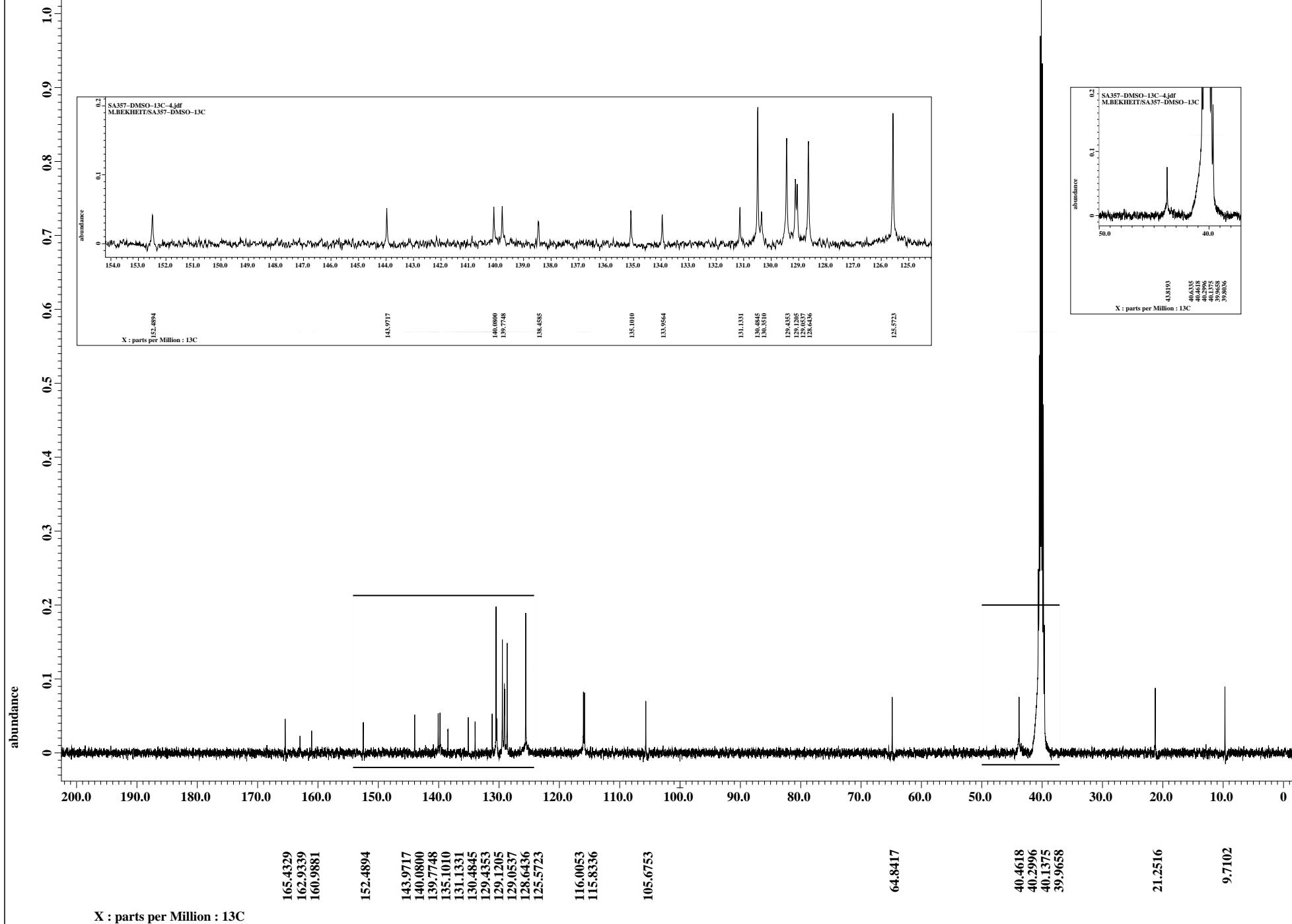

Supplement: Supplementary file 1 [file molecules-27-08904-s001.zip › Supplementary Materials/13 C NMR of 13.pdf]

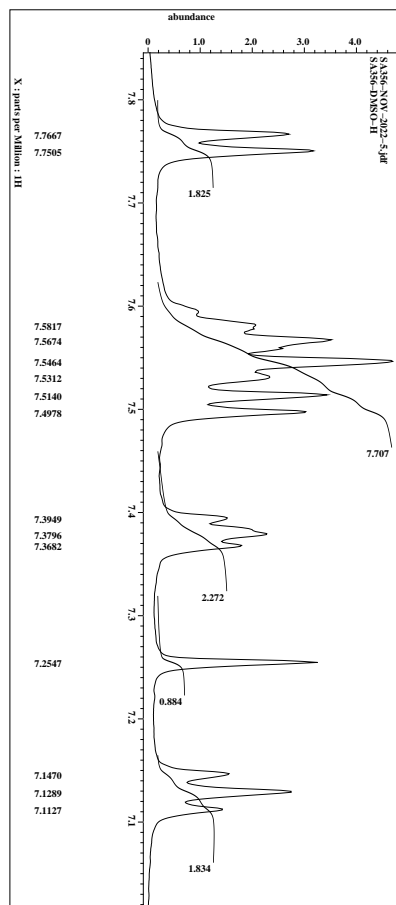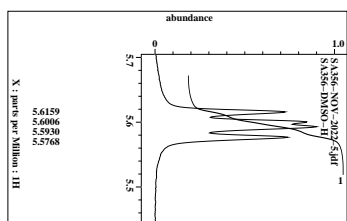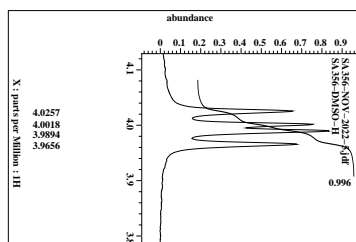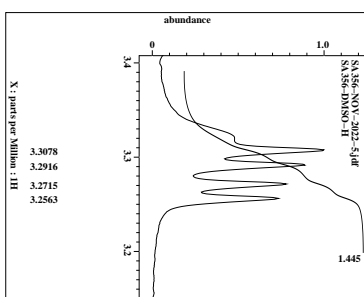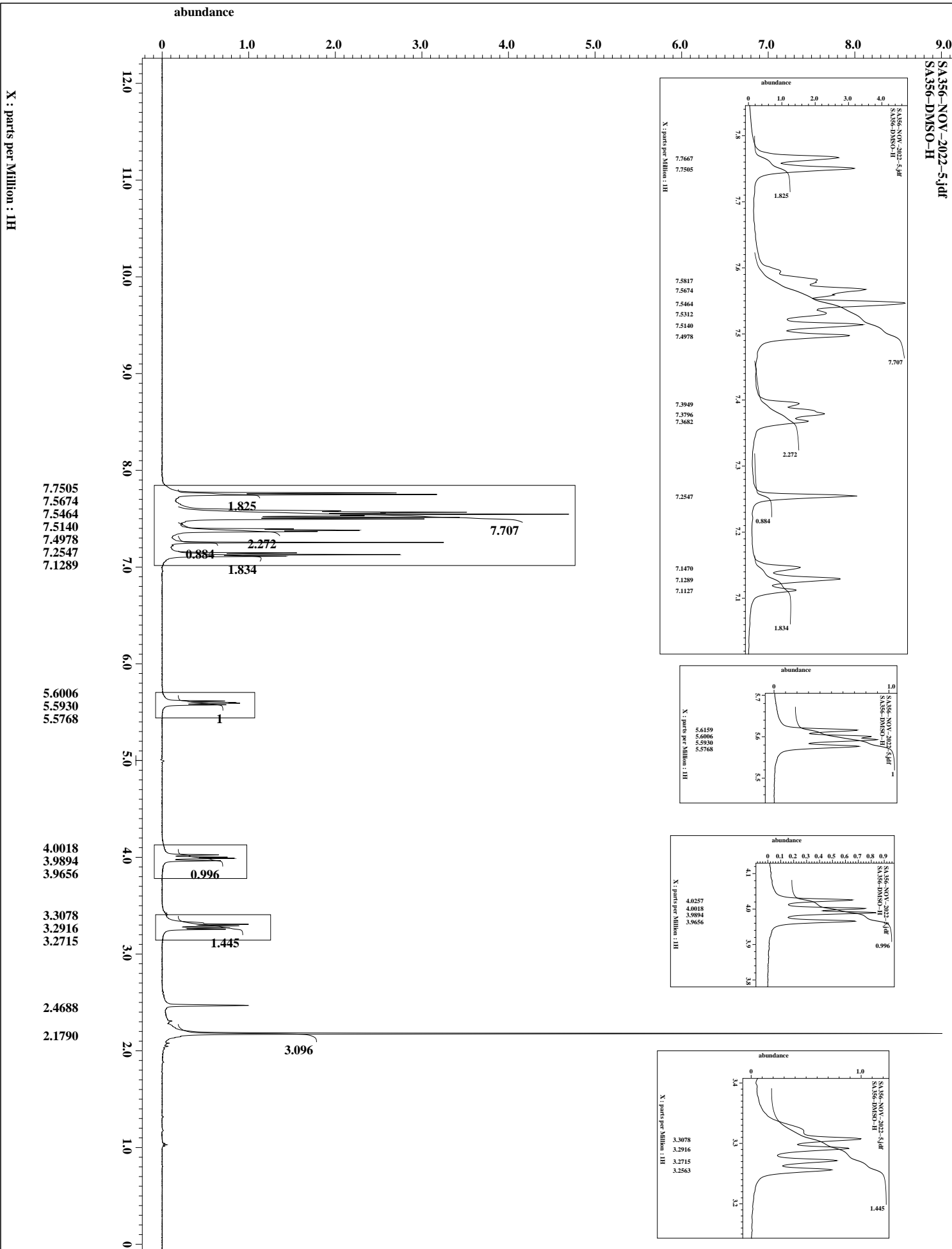

Supplement: Supplementary file 1 [file molecules-27-08904-s001.zip › Supplementary Materials/14 H NMR of 14.pdf]

SA356-DMSO-13C-4.jdf  
SA356-DMSO-13C

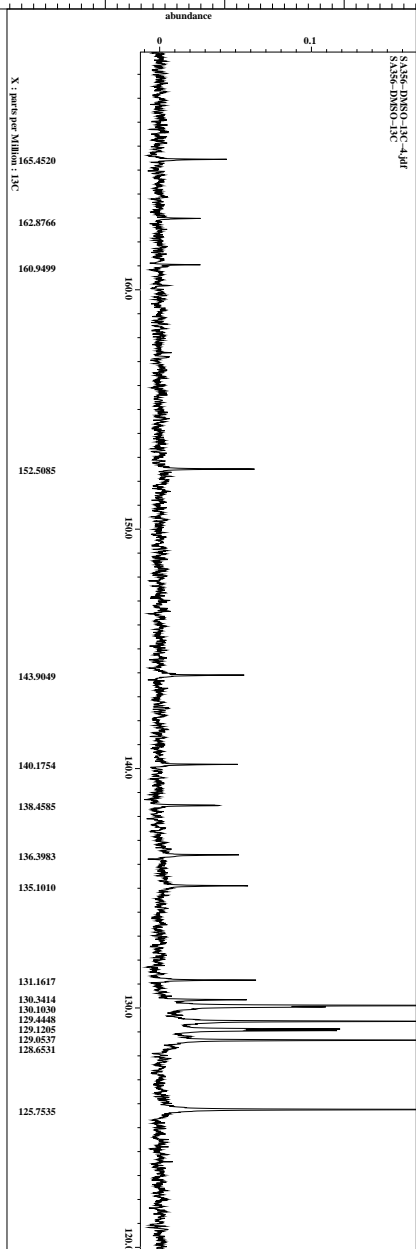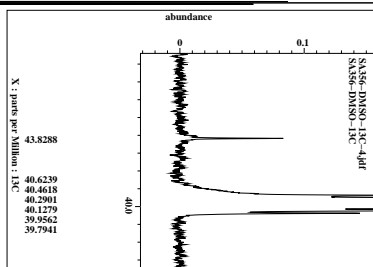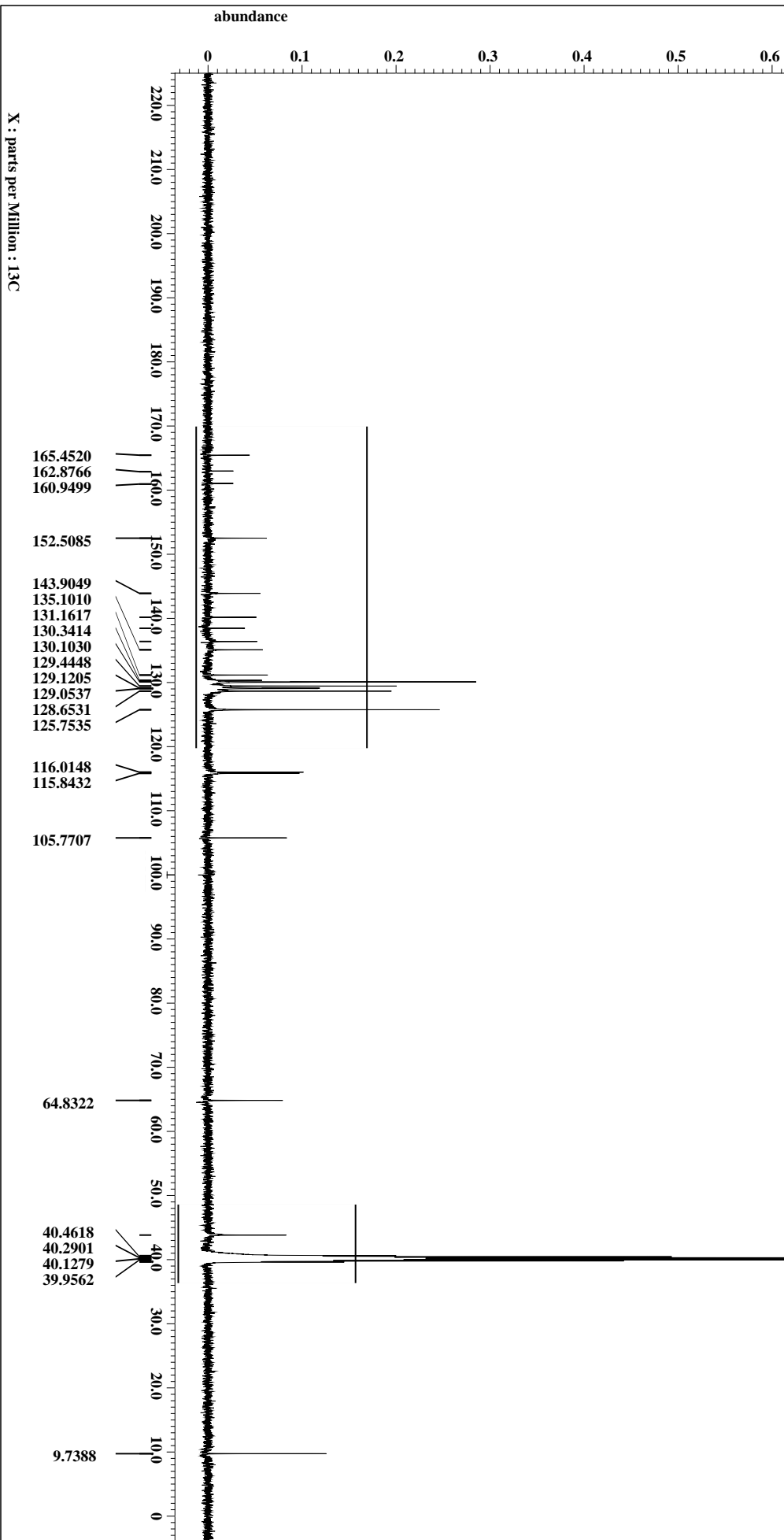

Supplement: Supplementary file 1 [file molecules-27-08904-s001.zip › Supplementary Materials/15 C NMR of 14.pdf]

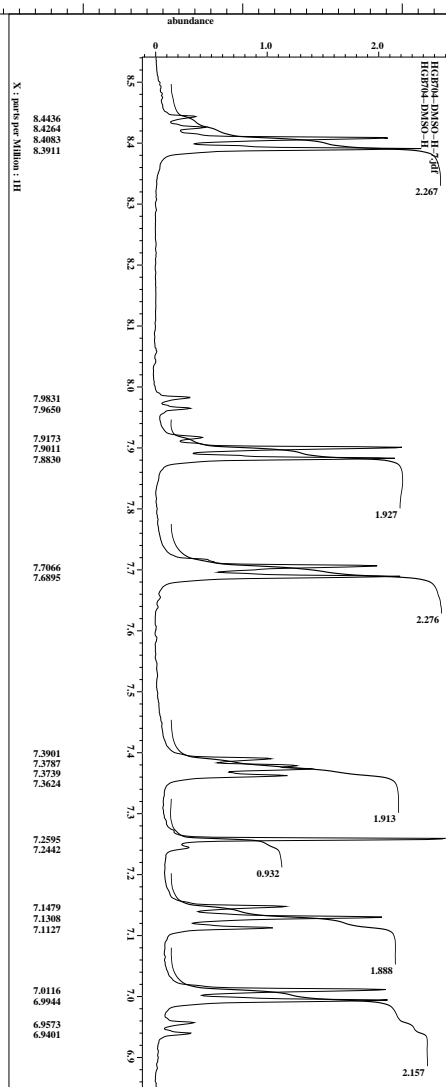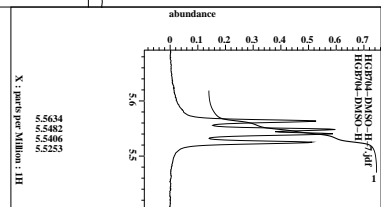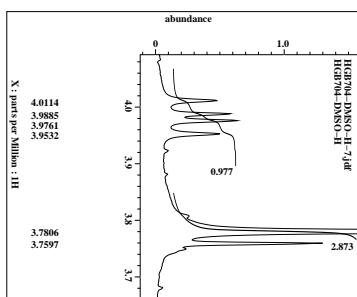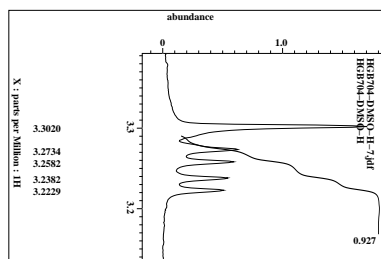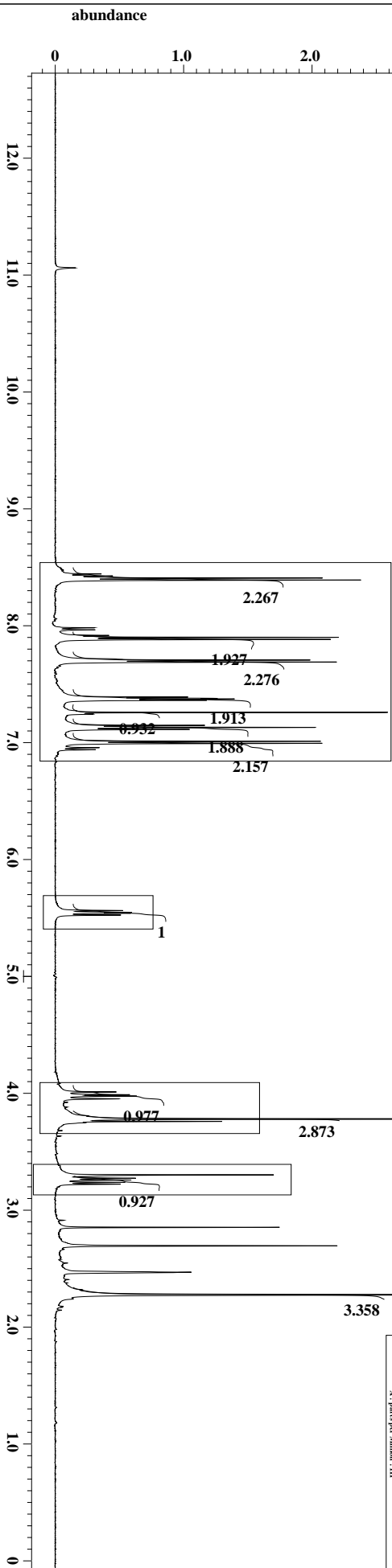

X : parts per Million : 1H

11.0615

8.4264  
8.4083  
8.3911  
7.9011  
7.8830  
7.7066  
7.6895  
7.3739  
7.2595  
7.1308  
7.0116  
6.9944

5.5634  
5.5482  
5.5406

3.9885  
3.9761  
3.7806  
3.7597

3.3020  
3.2734  
3.2582

2.8540  
2.6948  
2.4698  
2.2781

3.358

2.873

1

0.977

0.927

0.977

0.927

Supplement: Supplementary file 1 [file molecules-27-08904-s001.zip › Supplementary Materials/16 H NMR of 15.pdf]

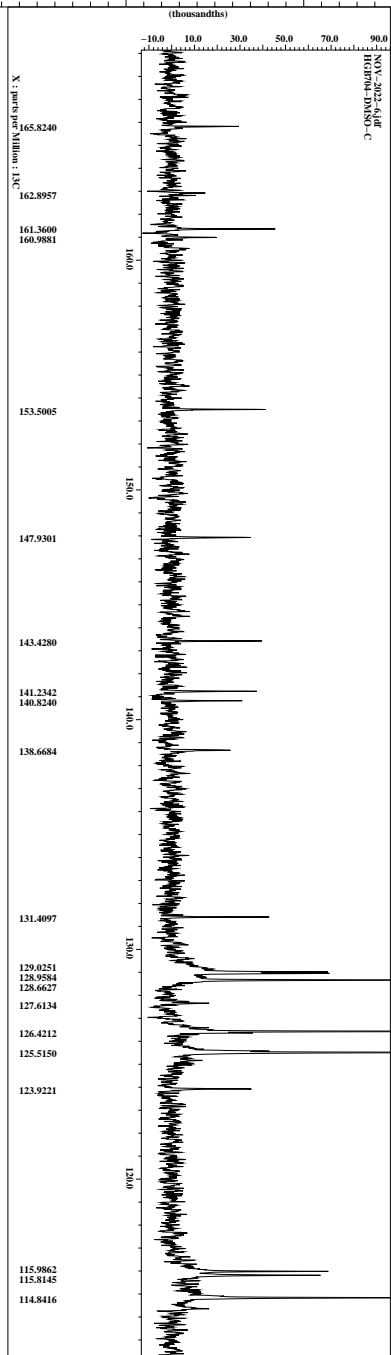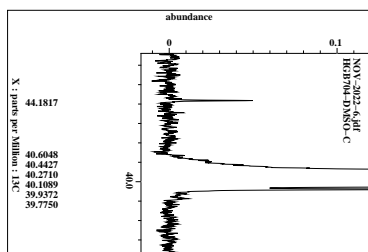

X : parts per Million : 13C

165.8240  
162.8957  
161.3600  
160.9881

153.5005

147.9301

143.4280

141.2342

140.8240

138.6684

131.4097

129.0251

128.9584

128.6627

126.4212

125.5150

115.9862

115.8145

114.8416

105.9233

64.5079

55.8852

40.4427

40.2710

40.1089

39.9372

9.8724

Supplement: Supplementary file 1 [file molecules-27-08904-s001.zip › Supplementary Materials/17 C NMR of 15.pdf]
